# Optimization of a tunable process for rapid production of calcium phosphate microparticles using a droplet-based microfluidic platform

**DOI:** 10.3389/fbioe.2024.1352184

**Published:** 2024-03-27

**Authors:** Y. Alaoui Selsouli, H. S. Rho, M. Eischen-Loges, V. P. Galván-Chacón, C. Stähli, Y. Viecelli, N. Döbelin, M. Bohner, Z. Tahmasebi Birgani, P. Habibović

**Affiliations:** ^1^ Department of Instructive Biomaterials Engineering, MERLN Institute for Technology-Inspired Regenerative Medicine, Maastricht University, Maastricht, Netherlands; ^2^ RMS Foundation, Bettlach, Switzerland

**Keywords:** calcium phosphates, water-in-oil emulsion, droplet microfluidics, in-droplet synthesis, inorganic additives, purification, sintering

## Abstract

Calcium phosphate (CaP) biomaterials are amongst the most widely used synthetic bone graft substitutes, owing to their chemical similarities to the mineral part of bone matrix and off-the-shelf availability. However, their ability to regenerate bone in critical-sized bone defects has remained inferior to the gold standard autologous bone. Hence, there is a need for methods that can be employed to efficiently produce CaPs with different properties, enabling the screening and consequent fine-tuning of the properties of CaPs towards effective bone regeneration. To this end, we propose the use of droplet microfluidics for rapid production of a variety of CaP microparticles. Particularly, this study aims to optimize the steps of a droplet microfluidic-based production process, including droplet generation, in-droplet CaP synthesis, purification and sintering, in order to obtain a library of CaP microparticles with fine-tuned properties. The results showed that size-controlled, monodisperse water-in-oil microdroplets containing calcium- and phosphate-rich solutions can be produced using a flow-focusing droplet-generator microfluidic chip. We optimized synthesis protocols based on in-droplet mineralization to obtain a range of CaP microparticles without and with inorganic additives. This was achieved by adjusting synthesis parameters, such as precursor concentration, pH value, and aging time, and applying heat treatment. In addition, our results indicated that the synthesis and fabrication parameters of CaPs in this method can alter the microstructure and the degradation behavior of CaPs. Overall, the results highlight the potential of the droplet microfluidic platform for engineering CaP microparticle biomaterials with fine-tuned properties.

## Introduction

The intrinsic ability of bone to regenerate after injury may be insufficient to heal large, critical-sized defects, for which bone grafting may be required (Winkler et al., 2018). The gold standard treatment of critical-sized bone defects, which is transplanting autologous tissue, i.e., a patient’s own bone, is only available in limited amounts and associated with a number of complications (Kubosch et al., 2016). This has led to a growing demand for (tissue) engineered bone graft substitutes that can effectively heal bone defects. In this context, bone graft substitutes based on synthetic biomaterials are of particular interest due to their off-the-shelf-availability and tunability (Damien and Parsons, 1991; Shah and Kamal, 2024). Calcium phosphate (CaP)-based biomaterials are amongst the most widely used synthetic bone graft substitutes, owing to their chemical similarities to the mineral part of the extracellular matrix of natural bone (Hou et al., 2022a; Mishchenko et al., 2023). In addition to being biocompatible, synthetic CaPs have been shown to induce osteogenic differentiation in different cell types *in vitro* (Barrère et al., 2006), and promote bone formation in orthotopic and sometimes ectopic sites *in vivo* (Habibovic et al., 2008; Yuan et al., 2010). Nonetheless, their ability to heal bone defects has remained inferior to the gold standard autologous bone. This is because, in contrast to autografts, not all CaP-based biomaterials are osteoinductive (Wang and Yeung, 2017; van Dijk et al., 2019; Xiao et al., 2020). The superior osteoinductivity of autografts is associated with specialized bone cells, growth factors, and a functional vascular network they contain. Tailoring physicochemical properties of CaPs, such as chemical composition, surface topography and microstructure, was shown to be effective in engineering CaPs with advanced osteoinductivity and bone-forming ability (Davison et al., 2014; Tang et al., 2018; van Dijk et al., 2023a; van Dijk et al., 2023b). This requires investigating a large design space to find the synthesis and processing parameters that lead to CaPs with optimal physicochemical properties that in turn result in effective biomaterials-induced bone healing.

One approach to design biomaterials with specific, desired interactions with biological systems is the rapid production and consequent screening of a large number of biomaterial formulations with varying physicochemical properties, often carried out in a miniaturized fashion to use minimal resources (Yang et al., 2021a). Such high-throughput production and screening approaches aim to find the optimal biomaterial synthesis/processing parameters and fine-tune the biomaterial properties towards the final application. Similar approaches have been commonly used in the past in the field of biopharmaceuticals (Bhambure et al., 2011). For designing instructive biomaterials, these approaches often exploit microtechnological tools to generate libraries or arrays of biomaterials (Vermeulen and de Boer, 2021), for example, with a variety of chemistries (He et al., 2011; Li et al., 2020) or surface topographies (Hulshof et al., 2017a; Hulshof et al., 2017b). Particularly microfluidic technologies are valuable tools for rapid and controlled production, as well as for screening of biomaterials (Kim et al., 2014; Guttenplan et al., 2021a). An example of microfluidic technologies in this context is droplet microfluidics, which has been used in the past to generate microparticles of various biomaterials (Long et al., 2023), including gelatin methacrylate, silk, poly (vinyl alcohol), and poly (lactic-co-glycolic acid) (Xu et al., 2009; Hou et al., 2018; Rho and Gardeniers, 2020; Han et al., 2021; Toprakcioglu and Knowles, 2021). Droplet microfluidic techniques offer several advantages over other standard particle production methods. An inherent property of all microfluidic-based methods is the reduced reagent consumption due to the small volume of fluids used for operation of microfluidic devices (Guttenplan et al., 2021a). Compared to bulk emulsion systems, the precise control over the fluid conditions in small volumes has rendered the droplet microfluidic-based techniques suitable for producing highly monodisperse particles (Guttenplan et al., 2021a; Cai et al., 2021; Seeto et al., 2022), from micro-to nano-scale (Gimondi et al., 2023; Nan et al., 2024). This also minimizes the batch-to-batch variation in terms of particle size. Additionally, generating multicomponent particles, for example, in core-shell or Janus forms (Huang et al., 2023a; Long et al., 2023; Wu et al., 2024), and to some extent, controlling particle shape (Cai et al., 2021; Huang et al., 2023a; Kittel et al., 2023; Long et al., 2023) can also be achieved with some droplet microfluidic systems. Another advantage lies in the ability of such systems to produce large numbers of individual microparticles per time unit (Guttenplan et al., 2021a). High-throughput microparticle production can be further enhanced by parallelizing multiple chips in one platform (Guttenplan et al., 2021a; Wu et al., 2021). Finally, using droplet microfluidics, precise doses of other compounds, such as drugs and growth factors, or cells can be encapsulated within the generated particles (Duncanson et al., 2012; Yang et al., 2021b; Huang et al., 2023b; Kieda et al., 2023; Zheng et al., 2023; Wang et al., 2024).

**FIGURE 1:**
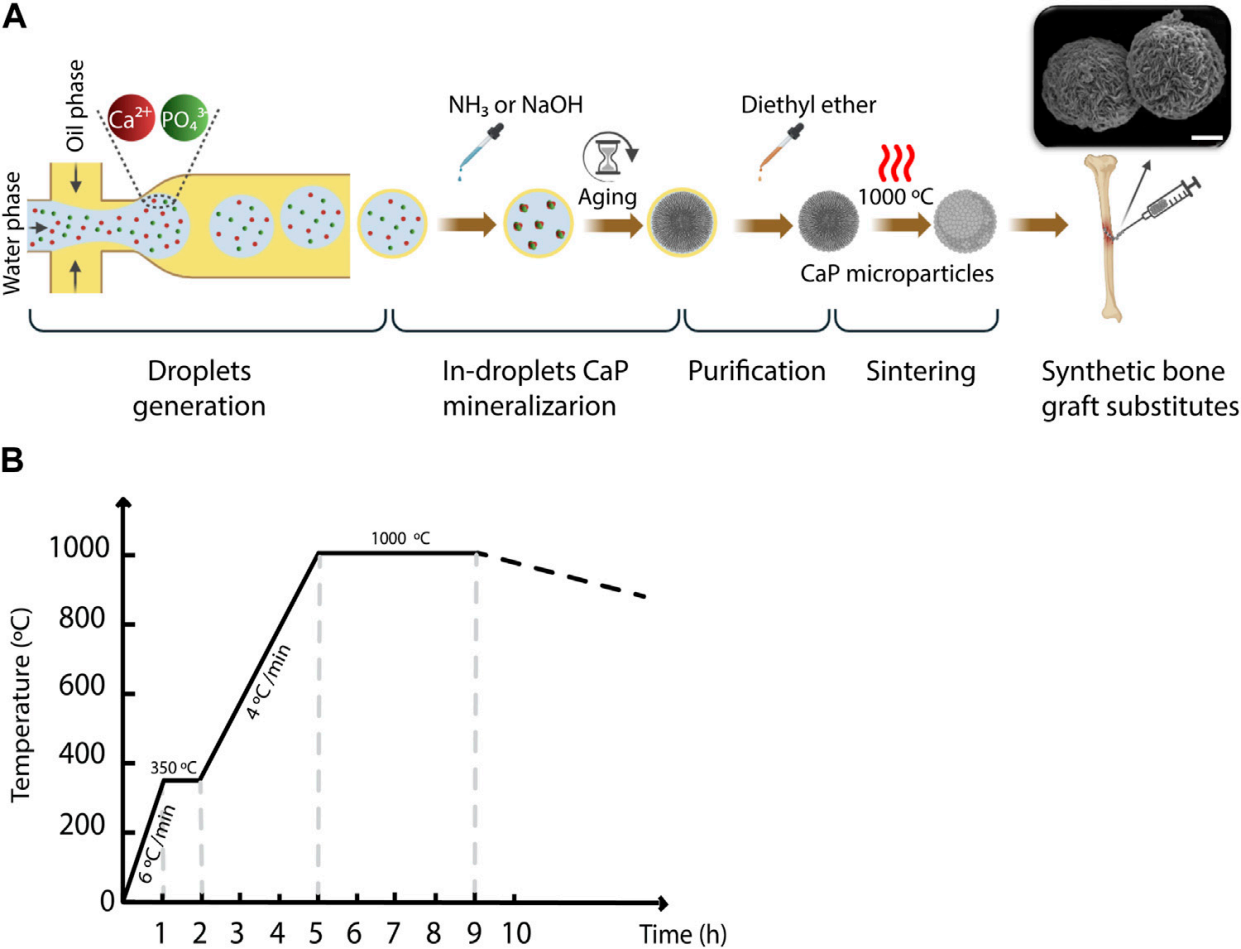
The workflow for generating CaP microparticles using the microfluidic droplet generator chip. **(A)** A schematic representation of the workflow used for the microparticle generation including droplet generation, in-droplet CaP mineralization, purification and sintering steps. Scale bar in SEM image: 10 µm. **(B)** Heat rate diagram of the sintering of CaP microparticles.

While droplet microfluidics has extensively been used for producing polymer/hydrogel microparticles, its use for producing inorganic CaP-based microparticles (Shum et al., 2009; Galván-Chacón et al., 2021) is scarce. Our group has previously applied a droplet-based microfluidic tool to synthesize CaP microparticles (Galván-Chacón et al., 2021). As a follow-up to this study, here, we aimed to optimize the CaPs production process using the droplet-based microfluidic platform to create a library of CaP microparticles with control over their properties. To achieve this, the processes of droplet generation and collection, in-droplet CaPs mineralization, purification, and post-fabrication sintering were optimized (Figure 1). The optimization steps aimed at generating monodisperse water-in-oil microdroplets with good stability over time to produce various CaP microparticles with controlled size. Furthermore, the potential of using this platform for adding different inorganic ions to the CaP microparticles was assessed, as it has been previously shown that inorganic ions can improve the bioactivity of CaPs while retaining their synthetic character (Tahmasebi Birgani et al., 2017). Finally, the physicochemical properties of different CaP microparticles and their degradation behavior in physiological conditions were assessed.

## Materials and methods

### Fabrication of microfluidic device

A glass-bonded polydimethylsiloxane (PDMS) microfluidic flow-focusing droplet generator was fabricated using standard soft lithography (Xia and Whitesides, 1998; Rho et al., 2021). In brief, the photomask design was prepared using CleWin software (WieWeb software) and printed on a 5-inch soda lime glass using an LBPG Heidelberg DWL200 mask generator (Heidelberg Instruments Mikrotechnik GmbH). A 4-inch silicon wafer (Si-Mat-Silicon Materials e.K.) was first dehydrated at 120°C for 10 min to increase photoresist adhesion to the silicon surface. A negative photoresist (SU-8 100, MicroChem Corp.) was then spun onto the silicon wafer at 2,500 rpm for 30 s to obtain a film thickness of 150 μm, and the wafer was pre-baked at 50°C for 10 min, 65°C for 30 min, and 95°C for 120 min. The photoresist-coated wafer was exposed to UV light through the photomask using a mask aligner (EVG 620, EV Group) at 12 mW cm^-2^ for 30 s. After post-exposure baking at 50°C for 10 min, 65°C for 10 min, and 80°C for 35 min, SU-8 structures on the wafer were developed by dip developing in propylene glycol monomethyl ether acetate (PGMEA, Sigma-Aldrich), followed by isopropyl alcohol (IPA) rinsing and blow-drying. Before PDMS replica fabrication, the wafer was hydrophobized with chlorotrimethylsilane (CTMS, Sigma-Aldrich) for 10 min in a desiccator to prevent the adhesion of PDMS. To replicate the SU-8 structures onto PDMS substrates, a degassed PDMS mixture (pre-polymer: curing agent = 10:1, RTV-615, Permacol BV) was poured onto the wafer and cured at 80°C for 45 min. Then, the PDMS layer was peeled off and cut, and holes for inlets and outlets were punched with a 25-gauge punch (Syneo Co.). The PDMS pieces and glass slides were plasma-treated (Femto PCCE, Diener) in oxygen at 0.3 mBar with a power of 70% for 30 s and bonded together. The resulting microfluidic droplet generators were placed in an oven at 80°C for 12 h in order to strengthen the PDMS-glass bond. The SU-8 mold and the PDMS replicates were inspected for the replication fidelity and height of the formed structures using a confocal laser scanning microscope-based profilometer (VK-X250, KEYENCE) integrated with MultiFileAnalyzer image analysis software (KEYENCE).

### Droplets generation, and production and post-processing of CaP microparticles

The oil phase, forming the droplet shells, consisting of a mixture of 10 wt% Span^®^ 80 nonionic surfactant (Sigma-Aldrich) and 90 wt% light mineral oil (Sigma-Aldrich), was filtered through a cellulose nitrate hydrophobic membrane (mesh size: 0.45 μm, Nalgene Rapid Flow, Fisher Scientific), and transferred to a 50-mL Falcon tube. The water phase, forming the droplet cores, consisted of mixtures of aqueous solutions of calcium nitrate tetrahydrate (99%, Ca(NO_3_)_2_·4H_2_O, Sigma-Aldrich) and phosphoric acid (80%, H_3_PO_4_, Sigma-Aldrich). By varying the mixing ratio of these solutions, starting calcium (Ca) to inorganic phosphate (P_i_) ion ratios (Ca/P) of 1, 1.5, and 1.67 could be obtained. To generate CaP microparticles with strontium (Sr) or zinc (Zn) addition, 10 at% of the Ca salt in the aqueous solution with a Ca/P of 1.67 was replaced by either strontium chloride (99%, SrCl_2_ · 6H_2_O, Sigma-Aldrich) or zinc chloride (99.99%, ZnCl_2_, Sigma-Aldrich). To generate CaP microparticles with both Sr and Zn incorporations, 20 at% of the Ca salt was replaced with a combination of Sr and Zn salts with equal at%. The aqueous solutions containing bivalent cations and P_i_ were mixed and filtered using a hydrophilic polyester membrane integrated within a bottle-top vacuum filtration system (mesh size: 0.2 μm, VWR) and transferred to a 50-mL Falcon tube. The inlets of the microfluidic chip were connected to 50-mL Falcon tubes containing the oil and water phases using Tygon^®^ Microbore tubes (Tygon^®^ ND-100-80, 0.020" IDX 0.060, Masterflex^®^) and dispensing metal tips (23 Gauge, 1/2", Nordson Benelux BV). The tubes with water and oil phases were in turn connected to pressure regulators (Precision pressure gauge, Festo) to control the flow rates of the solutions in the microfluidic channels. Here, by applying compressed nitrogen gas to the water and oil phases in the tubes, the liquids were pushed from the backside and guided towards the flow channels through the connecting tubes.

To generate the water-in-oil droplets, the pressure of the regulator of the oil phase was set to 0.4 bar, while that of the water phase varied between 0.14 and 0.24 bar, with a step size of 0.02 bar in order to vary the droplet volume. The outlet of the chip was connected to another Tygon^®^ tube to collect the droplets in a petri-dish. In the experiments designated for measuring the droplet size, a blue food dye (JO-LA) was used as the water phase to visualize the generated droplets. A microscope with a camera (SMZ25 stereomicroscope, Nikon), integrated with the set-up, was used to image the generated droplets (Figure 1A), and ImageJ software (LOCI, University of Wisconsin) was used to quantify the diameter of the droplets generated per condition. Either one or 2 M ammonia (NH_3_, 99.98%, Sigma-Aldrich), or 2 M sodium hydroxide (NaOH, 50% solution in water, Sigma-Aldrich) was added to the collected droplets, to increase the pH inside the droplets and induce the precipitation of CaPs. The CaP microparticles were gently separated from the suspension, and oil-purified by several filtration steps using cell strainers (mesh size: 100 μm, VRW) as filters and diethyl ether (≥99% stabilized, VWR) as a solvent for the oil phase (Figure 1B). In order to obtain CaP phases that are formed at elevated temperatures or have higher crystallinity, CaP microparticles were sintered at 1,000°C for 4 hours (Figure 1C). Table 1 summarizes the CaP microparticles included in the library, and their synthesis conditions and thermal post-processing.

**TABLE 1:** A summary of the library of the CaP microparticles, and their synthesis conditions and thermal post-processing (BS: Before sintering, AS: After sintering).

CaPs	(Ca + Sr + Zn)/P	Initial Ca concentration (M)	Initial P concentration (M)	Initial Sr concentration (M)	Initial Zn concentration (M)	Base type/concentration (M)	Aging time (h)	Sintering temperature (˚C)/Time (h)
CaP1-1M-BS	1	1.000	1.000	-	-	NH_3_/1	8	-
CaP1-0.5M-BS	1	0.500	0.500	-	-		12	-
CaP1-0.2M-BS	1	0.200	0.200	-	-		12	-
CaP1-1M- AS	1	1.000	1.000	-	-		8	1000/4
CaP1-0.5M-AS	1	0.500	0.500	-	-		12	1000/4
CaP1-0.2M-AS	1	0.200	0.200	-	-		12	1000/4
CaP1.5-BS	1.5	0.500	0.332	-	-	NH_3_/2 or NaOH/2	8	-
CaP1.5-AS	1.5	0.500	0.332	-	-		8	1000/4
CaP1.67-BS	1.67	1.000	0.600	-	-		8	-
CaP1.67-AS	1.67	1.000	0.600	-	-		8	1000/4
CaSrP1.67-BS	1.67	0.750	0.500	0.084	-		8	-
CaZnP1.67-BS	1.67	0.750	0.500	-	0.084		8	-
CaSrZnP1.67-BS	1.67	0.672	0.500	0.084	0.084		8	-

### Chemical composition and microstructure of CaP microparticles

To determine the CaP crystalline phases, CaP microparticles were analyzed with X-ray diffraction (XRD) at room temperature using an X-ray diffractometer (D2 PHASER, Bruker) with a copper (Cu) K_α_ radiation (wavelengths (λ) of 1.5406 Å), a diffraction angle (2θ) range of 6°–60°, a scan rate of 1°/min and a step size of 0.03°. The XRD patterns were analyzed using Profex 4.2.4, which allows identification and quantification of the crystalline phases in XRD patterns (Döbelin and Kleeberg, 2015). The chemical composition of the microparticles was further analyzed using attenuated total reflection Fourier-transform infrared spectroscopy (ATR-FTIR, Nicolet iS50, ThermoFisher Scientific) with the wavenumber range of 400–4,000 cm^-1^ and a step size of 0.482 cm^-1^.

The overall morphology and microstructure of the CaP microparticles were inspected using scanning electron microscopy (SEM, JSM-IT200, Jeol) at an accelerating voltage of 15–20 keV and magnifications of 500-10.000×. Prior to SEM imaging, CaP microparticles were mounted onto aluminum stubs using a double-sided carbon tape and coated with a thin layer of gold using a sputter-coater (SC7620, Quorum) in order to increase the conductivity of the surface.

The elemental composition of the CaP microparticles was first determined in the gold-sputtered samples using an energy-dispersive X-ray spectroscopy (EDS, JSM-IT200, Jeol) and then quantified further by inductively coupled plasma-mass spectrometry (ICP-MS; Agilent 7700x, Agilent Technologies). For the latter, 115 mg of the microparticles were dissolved in a mixture of one part of 69% nitric acid (HNO_3_) and four parts of 35% hydrochloric acid (HCl) and then diluted in a solution of demineralized water containing 3% HNO_3_, 2% HCl and 0.01% hydrofluoric acid (HF) (all: Rotipuran^®^ Supra, Carl Roth). ^44^Ca and ^31^P signals were calibrated against two custom-made certified standard solutions containing Ca and P ions at molar Ca/P ratios of either 1.0 or 1.5 (interpolated to match the Ca/P of each sample). ^66^Zn and ^88^Sr signals were measured and calibrated against a certified multi-element standard solution (all: Inorganic Ventures). Signal drifts were corrected, i), using an internal scandium (Sc) and in standard solution measured along with each sample (Inorganic Ventures) and, ii), by a continuous calibration correction based on the certified standard solutions measured after every 8^th^ sample. Moreover, interferences from Sr on the Ca signal were compensated based on the Ca signal measured in a Sr single-element standard solution (Inorganic Ventures). Finally, the mean values of four measurements per sample were determined.

### Porosity of CaP microparticles

To evaluate the effect of precursor concentration on the porosity of the microparticles, droplets with different concentrations of precursors (0.2, 0.5, and 1.0 M) and a Ca/P ratio of 1 were produced and mineralized as described above to obtain CaP microparticles. The resulting microparticles were also sintered as described above. The porosity measurements were performed on CaP microparticles tightly packed into circulars discs with diameters of 0.844 cm and heights of 0.12 cm (one disc per sample), and repeated 5 times per disc using a helium-pycnometer (Ultrapyc 1200e, Quantachrome Instruments). The percentage of the porosity (P%) in the CaP discs was calculated from the quotient of the apparent sample volume (Va) and the obtained pycnometric volume (Vp) using Eq. 1.

$$P\%=100\times \left(\text{Va}‐\text{Vp}\right)/\text{Va}$$ (1)

The porosity measurements described above consider the microporosity of the microparticles as well as that formed in between the particles within the CaP discs. To analyze the microporosity of the microparticles alone, we prepared 3 µm-thick sections of sintered microparticles obtained with different concentrations of precursors (0.2 and 1.0 M), which were then imaged by SEM. To that end, the microparticles were immersed in a resin (epon LX112, LADD) in a cone-shaped tube (BEEM capsule, Agar Scientific), and placed in an oven at 60^o^C for 3 days. The resulting block containing the microparticles were then sectioned using a diamond knife (Ultramicrotome, Leica EM UC7), carbon-sputtered, and imaged with SEM as described above. The fraction areas of the porosity of the microparticles were quantified in five regions of interest in the SEM images in binary mode using Fiji software.

### Surface roughness and profile of CaP microparticles

For each CaP, surfaces of at least three microparticles were scanned using a confocal laser scanning microscope-based profilometer at a magnification of ×150. For each microparticle, 4 squared regions (25 μm × 25 µm) were selected over the scanned surface and used for calculating the arithmetic mean heights of surface (Sa) (*n* ≥ 12) using the MultiFileAnalyzer image analysis software.

### Degradation of CaP microparticles

A degradations study was performed to evaluate the dissolution and precipitation of CaPs, and the consequent release or uptake of the inorganic ions. To evaluate the influence of the chemical phase on CaP degradation, CaPs with Ca/P of 1 and 1.5, before sintering, and 1.67, before and after sintering, and CaPs with the Sr and/or Zn addition were selected for this study. To determine the influence of the precursor concentration, we also included CaPs with a Ca/P precursor ratio of 1 with three precursor concentrations (0.2, 0.5, and 1 M). Triplicates of approximately 100 µL of CaPs were placed in a 24-well plate with 1 mL of cell medium composed of Minimum Essential Media (MEM Alpha 1X) without nucleotides and with L-Glutamax (Gibco) supplemented with 10% v/v fetal bovine serum (FBS, Lonza), 0.2 mM ascorbic acid (Sigma) and 100 Uml^-1^ Penicillin and 100 μg mL^-1^ streptomycin (Gibco), and incubated at 37°C, 100% humidity and 5% CO_2_. The media were refreshed every 2 days, up to 29 days, and collected for the analysis of the ion release/uptake at every media refreshment. Cell medium incubated at the same conditions without materials served as control. Ion concentrations were measured by ICP-MS (Agilent 7700x, Agilent Technologies). In short, medium aliquots were diluted 1:1000 in a solution of demineralized water containing 3% HNO_3_, 2% HCl and 0.01% HF, and ^44^Ca, ^31^P, ^66^Zn and ^88^Sr signals were measured and calibrated as described above. Finally, the mean values of three measurements of each of the three replicates of the medium samples were determined.

### Statistical analyses

The statistical analyses were performed in Graphpad v.9.3.0 (Prism) using a two-way analysis of variance (ANOVA) test followed by either Bonferroni (for the degradation of CaP microparticles) or Tukey (for the roughness and porosity of CaP microparticles) *post hoc* tests. Unpaired *t*-test was performed on the quantification of the porosity fraction areas using the SEM images of the sectioned microparticles. Statistically significant differences between different CaPs were plotted on the graphs and denoted as *, **, ***, and ****, indicating *p* ≤ 0.0332, *p* ≤ 0.0021, *p* ≤ 0.0002 and *p* ≤ 0.0001, respectively.

## Results and discussion

### Droplet generation, and CaP microparticles production and purification

**FIGURE 2:**
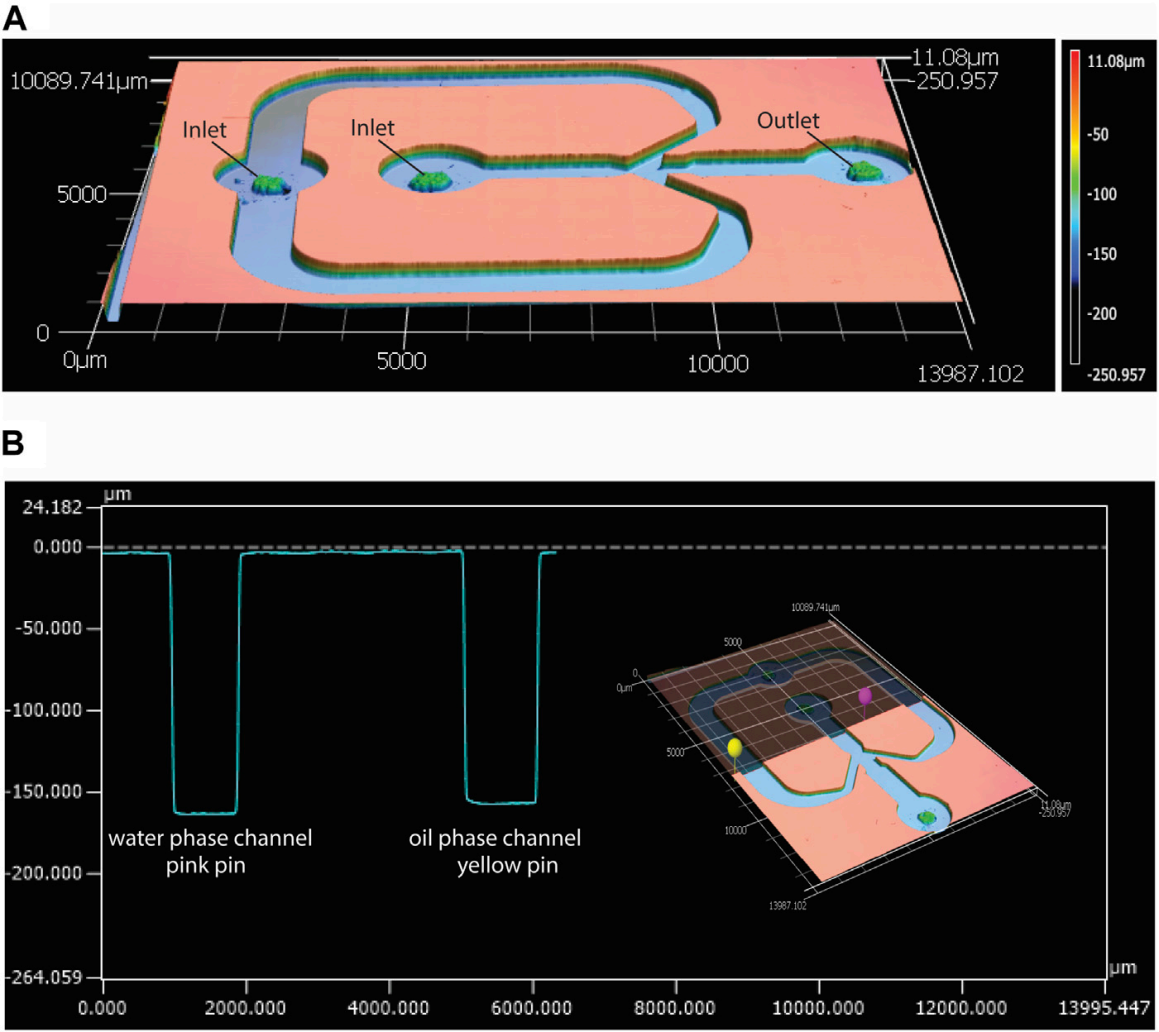
Characterization of the geometry of the microfluidic droplet generator chip with confocal laser scanning profilometer. **(A)** Height profile of the top PDMS piece of the microfluidic chip, and **(B)** an example of width and height measurements of the water phase (pink pin) and oil phase (yellow pin) channels in the PDMS piece, measured using the MultiFileAnalyzer software.

This study aimed to develop a standardized and easily-adjustable process for the production of a variety of CaPs in the form of microparticles. These biomaterials can later be screened for their osteogenic properties, and ultimately used for bone regeneration purposes. Examples of these applications include the development of CaP-based bone fillers, injectables or 3D scaffolds (van Dijk et al., 2019; Mofakhami and Salahinejad, 2021; Moussi et al., 2022; Ahlfeld et al., 2023). Moreover, CaP microparticles could also be used as reinforcing components in polymer matrices (Adhikari et al., 2021; Ahlfeld et al., 2023), for example, to produce composites with improved stiffness and bioactivity. A droplet-generating microfluidic chip was employed to create a library of CaP microparticles with fine-tuned physicochemical properties. This was achieved by introducing small modifications in the physical or chemical parameters that affect either the droplet generation, such as flow rate, chip geometry and dimensions, or CaP synthesis, such as concentrations of precursors and their ratio, aging time and post-processing treatments. A glass-bonded PDMS flow-focusing microfluidic chip was designed and fabricated, with the geometry shown in Figure 2A. The average width and height of the chip’s inner water phase channel were measured as approximately 930 ± 10 μm and 160 ± 1 μm, respectively. The average width and height of the outer, oil phase channel were respectively 1025 ± 5 μm and 160 ± 7 μm (Figures 2A, B). These results confirmed the fidelety of the PDMS replication method.

**FIGURE 3:**
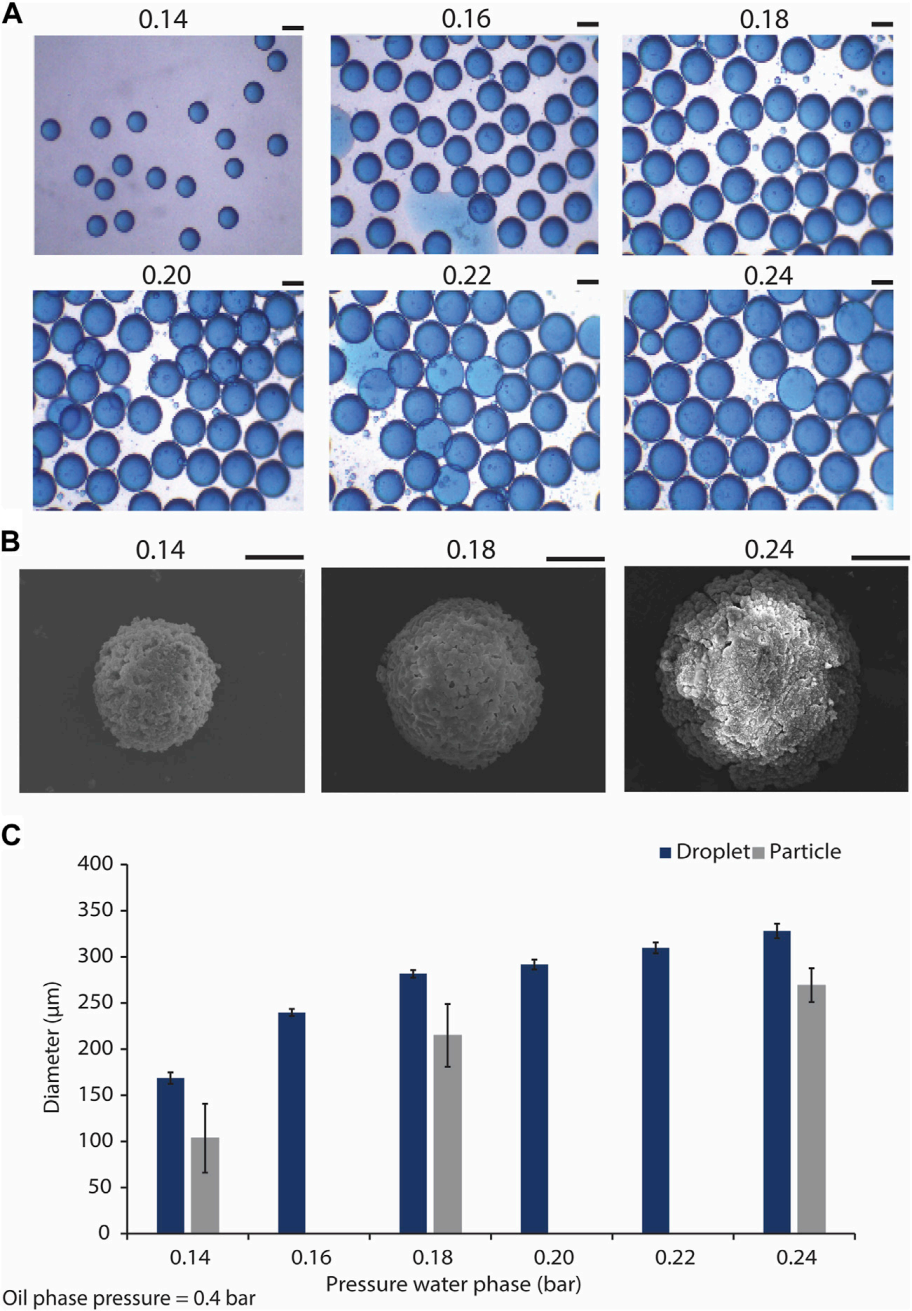
Generating microdroplets and CaP microparticles with difference sizes using the microfluidic droplet generator chip. **(A)** Droplets with different sizes, generated using the flow focusing microfluidic droplet generator, and by tuning the water phase pressure from 0.14 to 0.24 bar, and maintaining the oil phase pressure at 0.4 bar. Scale bars: 200 μm. **(B)** SEM images of CaP microparticles precipitated in droplets generated by water phase pressures of 0.14, 0.18, and 0.24 bar, and oil phase pressure of 0.4 bar. Scale bar: 50 μm. **(C)** Quantification of the diameters of the droplets (*n* = 22) and microparticles (*n* = 6) produced using the droplet generator at different water phase pressures.

We aimed to obtain monodisperse droplets with varying sizes by adjusting the flow rates of the water and oil phases using the pressure regulators integrated with the microfluidic setup. The results indicated that by maintaining the pressure of the oil phase at the constant value of 0.4 bars, and varying that of the water phase within the range of 0.14–0.24 bars, size-uniform droplets with average diameters of 169 ± 6 and 329 ± 8 μm were obtained for the lowest and the highest water phase pressures, respectively (Figures 3A, C; Supplementary Figure S1). This indicated an increase in the droplet diameter/volume by increasing the pressure of water phase. The presence of minute satellite droplets was however observed. These satellite droplets were not taken into consideration in the quantification of droplet size. The formation of satellite droplets has previously been described as a drawback of using flow-focusing droplet generators (Carrier et al., 2015). To overcome this issue, different solutions for the separation of satellite droplets from the main droplet population were suggested. For example, Tottori et al. coupled a flow-focusing device to a down-stream single-step deterministic lateral displacement (DLD) array resulting in separating the satellite droplets from the larger ones in a size-based approach (Tottori et al., 2017). However, in our study, we opted for down-stream size-based separation of CaPs that formed inside the satellite droplets with the size-defined filters used for the purification of the CaP microparticles from the oil phase.

CaP microparticles were synthesized in the microdroplets collected from the outlet of the microfluidic chips by the addition of a base solution to the droplets. This led to the diffusion of the base through the droplet oil shell into the water phase, resulting in an increase of the pH in the droplet core and precipitation of CaP microparticles, as described earlier (Galván-Chacón et al., 2021). CaP microparticles with average diameters of approximately 103, 215 and 269 µm were formed in the droplets obtained with the lowest, middle and highest water phase flow rates, respectively (i.e., respective water phase pressures of 0.14, 0.18 and 0.24 bars) (Figures 3B, C). These results indicated 39, 24% and 18% reduction in the diameters of the microparticles as compared to those of their corresponding droplets obtained with the lowest, middle and highest flow rates, respectively. In a similar approach, Galvan et al. obtained CaP microparticles that were approximately 35% smaller than the original droplets (Galván-Chacón et al., 2021), in accordance with our current observations. In-droplet synthesis of other inorganic microparticles has also been reported. Ermakov et al., for example, have recently generated monodisperse calcium carbonate micro- and nanoparticles using a droplet-generating microfluidic device, and used the resulting particles as carriers of a model peptide (Ermakov et al., 2024). It is known that producing CaP microparticles in the range of 100–300 µm using conventional particle production methods is challenging (Bohner et al., 2013). The ability to control the size of CaP microparticles, at different length scales, is an important feature of the presented biomaterial fabrication process, as it enables the fabrication of CaPs that are suitable for various types of *in vitro* and *in vivo* biological testing and applications. More importantly and in the context of biological performance, it has been previously shown that particle size can influence the cell and tissue response to CaPs. For example, Mankani et al. have demonstrated that when loaded with human bone marrow stromal cells (hBMSCs), HA/TCP microparticles of 0.1–0.25 mm in diameter showed a more pronounced bone formation in 4- and 10-week subcutaneous implantations in mice as compared to microparticles with smaller or larger particle sizes (Mankani et al., 2001). Similarly, Balaguer et al. showed that biphasic calcium phosphate (BCP) microparticles of 80–200 μm, consisting of HA and TCP phases, embedded in blood clot induced more ectopic bone formation, i.e., exhibited osteoinduction, as compared to the implants made with BCP microparticles with diameters of 40–80 or 200–500 μm, in which no ectopic bone formation was observed (Balaguer et al., 2010). Wang et al., confirmed the effect of microparticle size on osteoinductivity of BCPs, showing ectopic bone formation in BCP microparticles only when they are larger than 45 µm (Wang et al., 2015). It has also been suggested that CaP microparticles with multiple fine-tuned granulometries can control the bone formation process at different stages of defect healing, with different populations of microparticles fulfilling different functions (Baroth et al., 2009). This information highlights the significance of particle size and size range for achieving desirable clinical outcomes with CaP microparticles, which can be easily tailored with the droplet microfluidic-based fabrication process described here.

**FIGURE 4:**
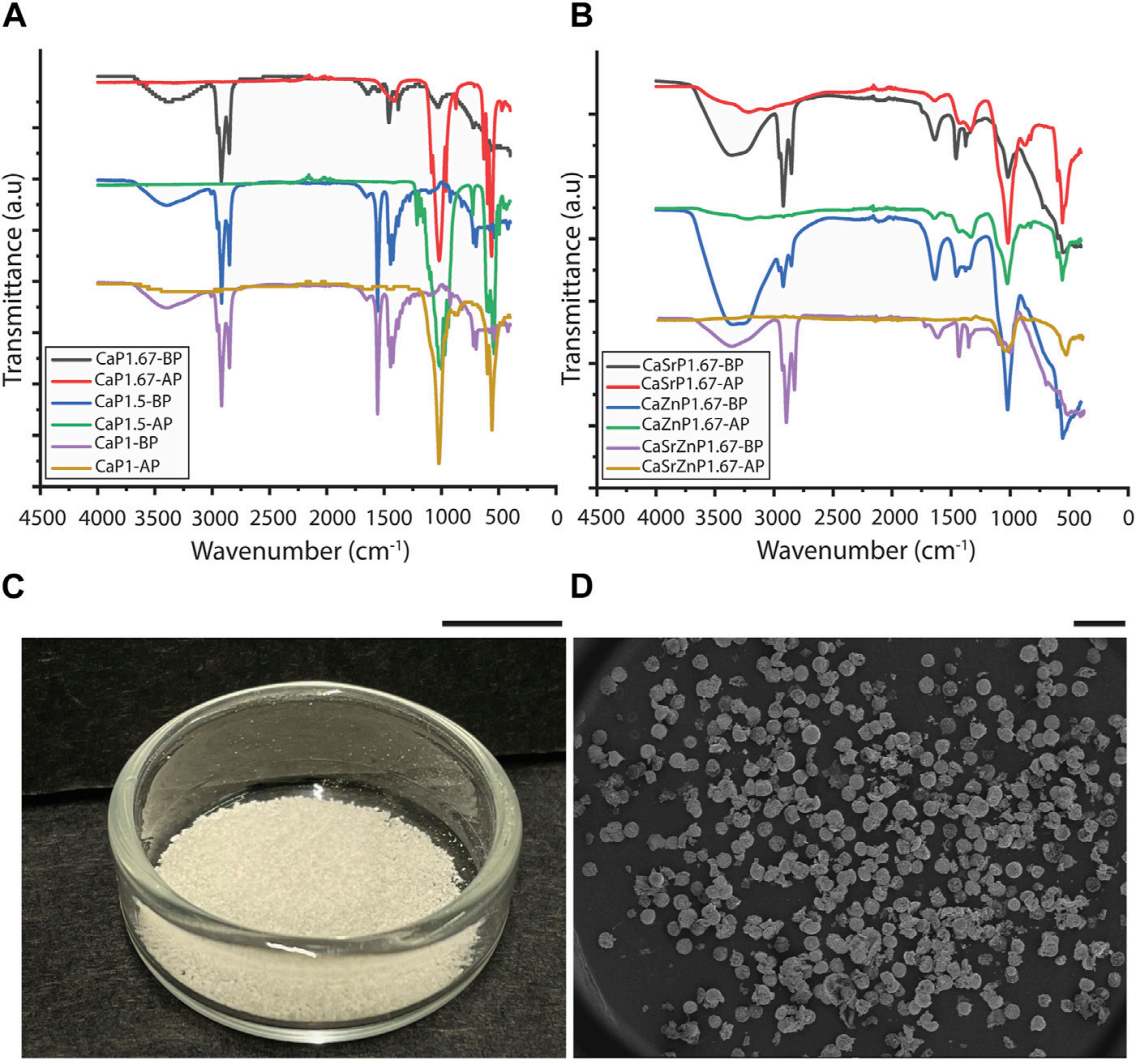
Purification of CaP microparticles. FTIR spectra of different CaP microparticles **(A)** without and **(B)** with the addition of inorganic ions before purification (BP) and after purification (AP). **(C)** CaPs microparticles obtained from one round of synthesis. Scale bar: 1 cm. **(D)** SEM image showing the overall shape of the produced CaP microparticles. Scale bar: 500 μm.

To evaluate the efficiency of the purification process, FTIR was performed on the suspensions containing CaPs before purification (CaPs-BP) and the CaPs after purification (CaPs-AP) (Figures 4A, B). The FTIR spectra of the CaPs-BP showed strong triple bands at around 2,850-2,960 cm^-1^ and the bands at around 1,375-1,550 cm^-1^, corresponding to the stretching and bending of C-H bond, indicating the presence of mineral oil (Hadialnashia et al., 2018; Kurzweil et al., 2021). In addition, some of these bands have been previously seen in Span^®^ 80, too (Fu et al., 2015; Kavita et al., 2016). The broad band at 3398.5 cm^-1^ corresponds to the stretching of O-H groups, which could be due to the presence of water and Span^®^ 80 in the samples before purification, and disappeared after purification and drying of the samples. In the FTIR spectra of CaPs-AP, the typical bands corresponding to PO_4_^3-^ and HPO_4_^2-^ groups became visible at around 560-620 and 1,000-1,100 cm^-1^. The bands attributed to the mineral oil were, in most cases, absent in CaPs-AP. This indicated the effective removal of the oil phase during the purification step. Occasionally, these bands appeared in the FTIR spectra of CaPs-AP, but with substantially lower intensities as compared to those in CaPs-BP, plausibly due to the presence of small residues of the oil phase. In CaP1.67-BS-AP without additional inorganics, and possibly with individual and to a lesser extent, combined incorporation of Sr and Zn, CO_3_^2-^ bands at around 870 and 1,420 cm^–1^ were detected (Chandrasekaran et al., 2013; Lawton et al., 2015), indicating the presence of a carbonated CaP phase.

Various techniques have been used to remove the oil phase from microparticles fabricated using emulsion techniques. In previous reports, thermal post-treatments resulted in the removal of remaining oil from CaP particles synthesized using emulsions methods (Chen et al., 2009; Shum et al., 2009). However, as we aimed to generate microparticles of thermally less stable CaP phases too, we opted for using an organic solvent that removes the oil, in this case diethyl ether (Juhaimi et al., 2019). Diethyl ether is non-polar and hence, facilitates the removal of non-polar mineral oil. In addition, it is volatile and allows the rapid evaporation of any residue after oil removal. Similar to our approach, other organic solvents, including methanol (Singh et al., 2008), cyclohexane in acetone (Galván-Chacón et al., 2021), and isopropanol (Hou et al., 2022b), were also previously used to remove the oil residue from CaP-based materials.

In all conditions, the CaP microparticles assumed a (semi-)spherical shape during precipitation, and in spite of their inherent brittleness (Jeong et al., 2019), they preserved this shape during purification (Figures 4C, D). The shape of inorganic microparticles is also known to influence the corresponding cellular responses (Parakhonskiy et al., 2015; Sridharan et al., 2020). However, this study only focused on controlling the particle size, chemistry, and microstructure.

### Chemical composition and microstructure of CaP microparticles

**FIGURE 5:**
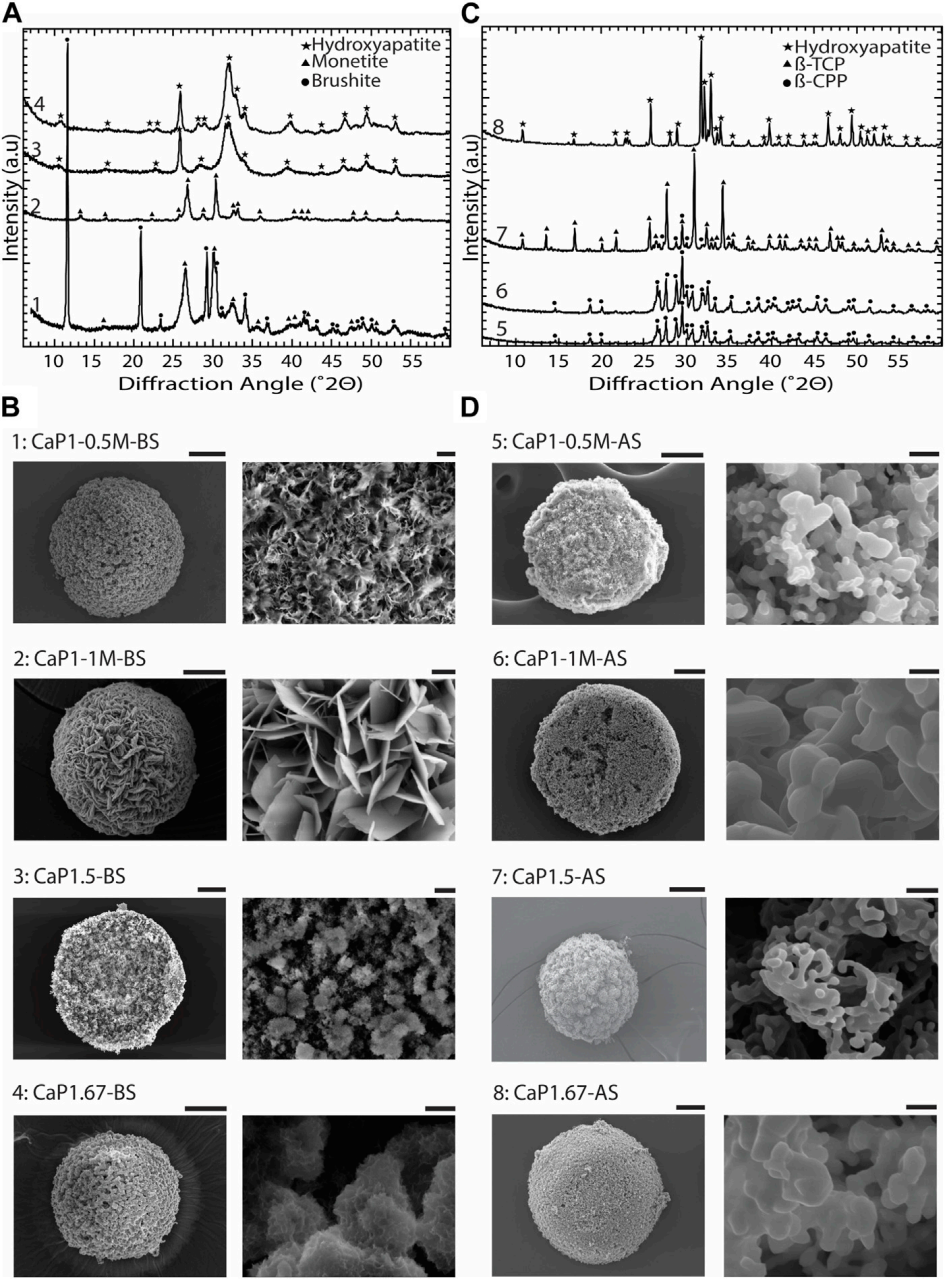
Chemical composition and microstructure of CaPs. **(A)** XRD patterns and **(B)** SEM images of CaP microparticles without the addition of inorganic ions before sintering (BS). **(C)** XRD patterns and **(D)** SEM images of CaP microparticles without the addition of inorganic ions after sintering (AS). Scale bars for the SEM images of microparticles [left column in **(B,D)**]: 50 μm, and for the zoomed images of the microstructure of microparticles [right column in **(B,D)**]: 2 μm.

To obtain different CaP phases, the CaP synthesis parameters and sintering were varied (Table 1). XRD patterns and imaging results confirmed that by varying the Ca/P of the pressure solution, the initial precursor concentrations, pH levels, and aging time, and by applying sintering, CaP microparticles with different chemical phases and distinct microstructures could be obtained (Figure 5).

In CaP1-0.5M-BS with 12 h of aging, a combination of dicalcium phosphate dihydrate (DCPD, brushite), and dicalcium phosphate anhydrous (DCPA, monetite), was formed, with brushite being the predominant phase. In this case, the CaP presented a microstructure composed of submicron crystals. However, in a similar Ca/P ratio with higher initial precursor concentrations and a reduced aging time of 8 h (CaP1-1M-BS), pure monetite, with a crystalline microstructure composed of micron-sized plates was formed (Figures 5A, B). After sintering, both CaPs formed at a Ca/P of 1 (i.e., CaP1-1M-AS and CaP1-0.5M-AS) transformed to β-pyrophosphate (β-CPP) (Figure 5C) with grained morphologies (Figure 5D). Previous studies also reported the formation of brushite and monetite at a Ca/P of 1 (Boanini et al., 2021; Zhou et al., 2021), and their transformation to β-CPP at elevated temperatures (Maity et al., 2011; Anastasiou et al., 2016). For example, Maity et al. used a reverse microemulsion system to produce nanocrystalline brushite particles with needle-like morphology, which also turned to a grained CPP after sintering at 800°C (Maity et al., 2011). Previously, several dry and wet synthesis methods were reported to form monetite (Zhou et al., 2021), amongst which obtaining monetite through dehydration of brushite has often been reported in the literature (Ma et al., 2006; Dosen and Giese, 2011; Galván-Chacón et al., 2021). In our system, however, monetite phase was formed at room temperature by increasing the concentration of precursors as compared to the conditions used for synthesizing brushite. This may be related to the lower pH in the solution with higher concentration of precursors, as it was previously indicated that in acidic conditions, brushite can transform to monetite at low temperatures (Bohner et al., 2000). Leeuwenburg et al. could also obtain a crystalline monetite coating using 0.05 M Ca and Pi precursor solutions without additional heat treatment via an electrostatic spray deposition method. This was the highest precursor solution concentration in this study, where lower precursor concentrations led to the formation of amorphous CaP (Leeuwenburgh et al., 2004). Our results therefore confirm that in addition to Ca/P ratio, the concentration of Ca and Pi precursor solution could impact the chemical composition of the resulting CaP phases.

XRD analyses of CaP1.5-BS and CaP1.67-BS microparticles presented the typical pattern of hydroxyapatite (HA) with relatively broad peaks (Figure 5A), indicating the low crystallinity of the formed HA in both conditions. In both cases, the obtained HA phases exhibited fine, clustered, submicron structures (Figure 5B). In CaP1.5-BS, the structures appeared more needle-like and formed smaller clusters, while in CaP1.67-BS, the structures resembled a flaked morphology and formed larger clusters. Sintering of low crystalline HA with the Ca/P of 1.67 (CaP1.67-AS) allowed the formation of a highly crystalline phase predominantly made of HA (Figure 5C) with a grained microstructure (Figure 5D). Similar observations were reported in different methods of synthesizing HA. For example, Rodriguez-Lugo et al. used a wet chemical method to synthesize a low crystalline HA in an aqueous solution and reported a pH- and temperature-dependent HA microstructure. Furthermore, similar to our results, they observed a grained morphology in the HA phase after sintering (Rodríguez-Lugo et al., 2018). In our study, the formation of β-tricalcium phosphate (β-TCP) (CaP1.5-AS, Figure 5C) with a grained morphology (Figure 5D), as the main phase, was achieved by sintering of low-crystalline HA with the Ca/P of 1.5. Different methods for producing β-TCP have been reported in the literature, for example, solid-state reaction between Ca- and Pi-rich phases (Jinlong et al., 2001), precipitation in organic media (Tao et al., 2008) and thermal conversion of other (low temperature) CaPs, such as amorphous calcium phosphate (ACP) (Maciejewski et al., 2008) or calcium deficient hydroxyapatite (CDHA) (Liou and Chen, 2002), the latter being in line with our results.

Overall, these results indicate that controlled and efficient production of different CaPs can be achieved using in-droplet synthesis through manipulating the synthesis parameters and applying thermal post-treatment. In optimizing the synthesis protocols, lack of specific analytical tools to monitor the kinetic of in-droplet synthesis of the CaPs presented a challenge. This was particularly highlighted in monitoring and controlling the internal pH of the droplets throughout the CaP synthesis process, which led to a ‘black box testing’ approach for optimizing the mineralization protocols. *Rana* et al., proposed the control of in-droplet pH over time by using double emulsion droplets, with the enzyme urease encapsulated in the core (Rana et al., 2023). Urease can produce ammonia and carbon dioxide within the droplets through hydrolysis of urea, creating a feedback loop for controlling the pH. By using this pH-regulating system, in-droplet synthesis of HA and brushite was demonstrated. For future research, use of such pH regulating systems, or development and use of reliable and sensitive pH indicators compatible with emulsion systems, for example, molecular pH sensors (Steinegger et al., 2020), is suggested.

### Chemical composition and microstructure of CaP microparticles with inorganic additives

Doping or loading CaP biomaterials with inorganic additives, such as magnesium (Mg), strontium (Sr), copper (Cu) and zinc (Zn), that are present in bone matrix in trace amounts (Gaffney-Stomberg, 2019) and are often known for their therapeutic purposes (Tahmasebi Birgani et al., 2017; Chetan, 2022), is realized as a promising strategy to improve the regenerative capacity of the CaPs while maintaining their synthetic characteristics, long half-life and low production costs. These inorganic additives are known to play substantial roles in biological mechanisms involved in bone homeostasis and regeneration (Birgani et al., 2016a; Šalandová et al., 2021; Sutthavas et al., 2022) and to influence characteristics of bone mineral, such as its hardness (Noviyanti et al., 2021), compressive strength (Bose et al., 2011), and crystallinity (Garbo et al., 2020).

For example, Zn in ionic form was shown to promote cell proliferation and osteogenic differentiation, by stimulating alkaline phosphatase (ALP) activity, an early marker of osteogenic differentiation, and collagen synthesis in murine pre-osteoblastic MC3T3 cells (Seo et al., 2010). It is known that the concentrations of inorganic ions in these cases are determinant of the cell response. For example, Yu et al. demonstrated that while low concentrations of Zn^2+^ (below 5 μg/mL) led to enhanced osteogenic differentiation of rat bone marrow-derived mesenchymal stem cells (rBMSCs), higher Zn^2+^ concentrations in cell media (15 μg/mL) reduced rBMSC adhesion and viability and suppressed their osteogenic differentiation (Yu et al., 2020). Zn is also a known inhibitor of osteoclastic mineral resorption (Moonga and Dempster, 1995), and was shown to suppress differentiation of RAW264.7 osteoclasts when supplemented in cell media (Yamaguchi and Weitzmann, 2011) and to reduce osteoclastic resorption activities when administered orally to rats (Hadley et al., 2010). In addition, evidence for antibacterial effects of Zn, when incorporated into bioceramics, was reported before (Hu et al., 2012). Sr is another inorganic additive often used to improve the biological performance of CaPs. Sr, being chemically similar to Ca, is known to play a dual role in bone regeneration by promoting bone formation while suppressing bone resorption. In fact, in the form of ranelate salt, Sr is clinically used as an anti-osteoporosis drug (Meunier et al., 2004; Kołodziejska et al., 2021). Oral administration of strontium ranelate was previously shown to improve the bone mass and strength in rats (Kołodziejska et al., 2021) and the bone mineral density in postmenopausal women with osteoporosis (Meunier et al., 2004). Incorporation of Sr into CaP scaffolds was reported to increase the compressive strength of the scaffolds and to improve the viability and ALP level in rBMSCs cultured on the scaffolds (Li et al., 2021). Similarly, increased proliferation and expression of multiple osteogenic biomarkers in human bone marrow-derived mesenchymal stromal cells were observed when cultured on Sr-incorporated CaP coatings (Birgani et al., 2016b). Based on the proven effects of Sr and Zn on osteogenesis-related processes, here, droplet microfluidics-based process flow was used to also generate CaP microparticles containing these two inorganic additives. To that end, during the fabrication of CaP1.67-BS, either 10 at% of the Ca precursor was substituted with Sr or Zn precursors, or 20 at% of the Ca precursor was substituted with a combination of Sr and Zn precursors (with equal at%).

**FIGURE 6:**
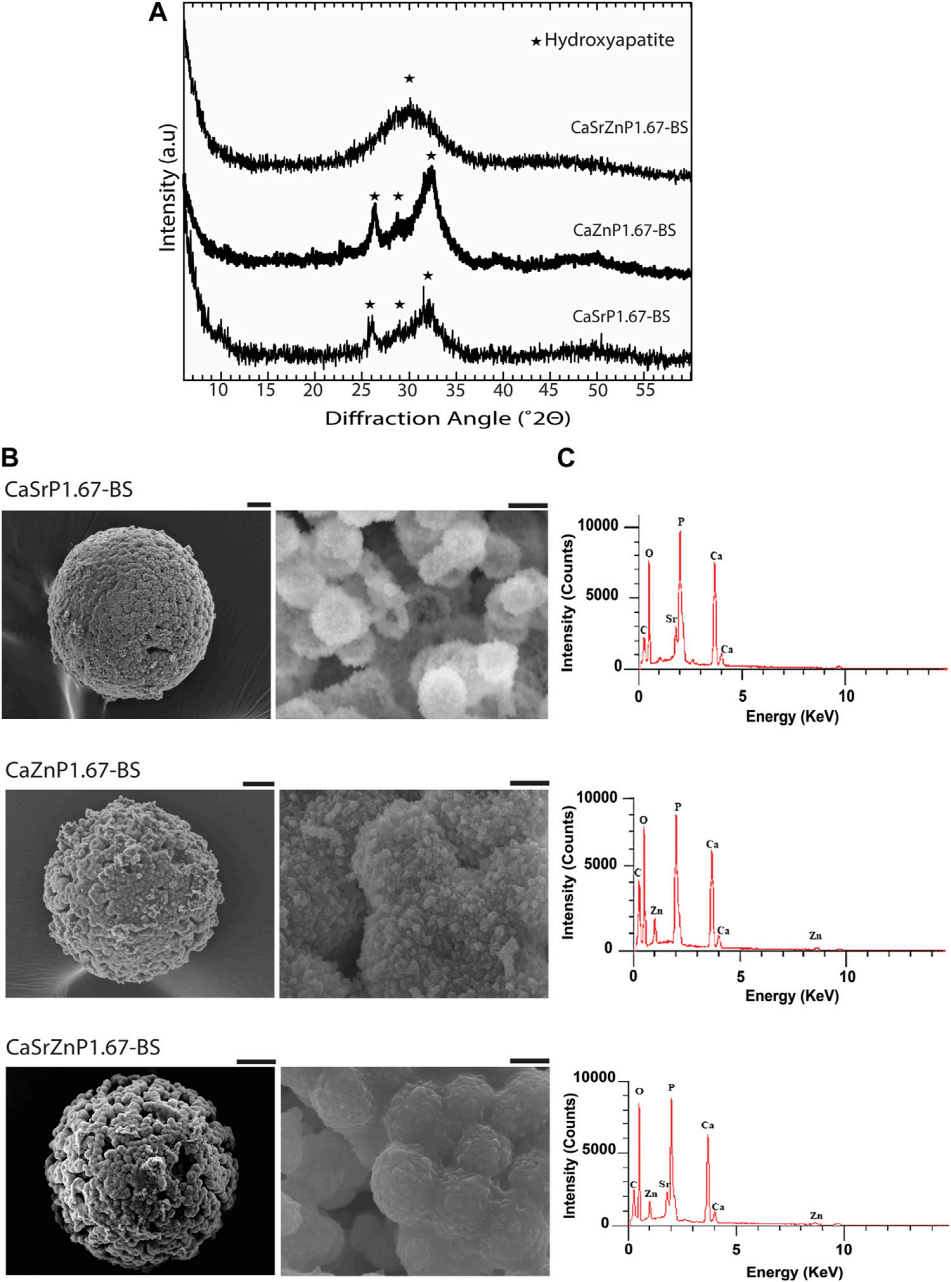
Chemical composition and microstructure of CaPs with inorganic additives. **(A)** XRD patterns and **(B)** SEM images of CaP microparticles with inorganic ions before sintering (BS). Scale bars for the SEM images of microparticles [left column in **(B)**]: 20 μm, and for the zoomed images of the microstructure of microparticles (right column in b): 2 μm. **(C)** EDS spectra of CaP microparticles with the addition of inorganic ions.

The XRD patterns of the resulting microparticles in all three cases indicated the presence of HA, with lower crystallinity as compared to CaP167-BS in case of individual addition of Sr or Zn, i.e., CaSrP1.67-BS or CaZnP1.67-BS (Figure 6A; Supplementary Figure S2). An amorphous apatitic phase was obtained in case of dual addition of Sr and Zn, i.e., CaSrZnP1.67-BS (Figure 6A). In line with these observations, Li et al. previously showed that by increasing Sr content in an Sr-incorporated apatite, the crystallinity and size of the crystals were altered (Li et al., 2007). Similarly, Miyaji et al. observed that the peaks in the XRD patterns of Zn-incorporated apatite became broader by increasing the Zn molar fraction from 0 to 15 mol.% in the precursor solutions during the synthesis, indicating a decrease in the crystallinity of the apatite phase by increasing Zn substitution (Miyaji et al., 2005). SEM analysis of CaSrZnP1.67-BS also showed less morphologically defined microstructures (Figure 6B) as compared to HA without ion addition, in accordance with our previous study (Galván-Chacón et al., 2021) and similar to that in previously reported low-crystalline apatites (Drouet, 2013). It has been shown that the changes in the CaP morphology are influenced by the incorporation dose too. For example, Yang et al. showed that the Zn addition at low levels had no effect on the CaP morphology, while high Zn levels increased the size of the crystals. In the same study, the Sr incorporation with different concentrations was reported not to affect the morphology (Yang et al., 2010). In contrast, Birgani et al. observed changes in crystal morphology by incorporating Sr into CaP coatings, an effect that was dependent on the incorporation dose (Birgani et al., 2016b).

EDS spectra of the CaP microparticles with ion addition indicated the presence of the ions (Figure 6C). Quantification of the EDS results indicated a Sr or Zn content (per total cation content) of 11.5 at% in CaSrP1.67-BS, 10.8 at% in CaZnP1.67-BS, and 9.8 at% of Sr and 9.1 at% of Zn in CaSrZnP1.67-BS (Supplementary Table S1). However, due to the semi-quantitative nature of EDS analysis, the exact amounts of the inorganic additives in CaPs were determined using ICP-MS, where the results indicated a Sr or Zn content (per total cation content) of 9.3 and 10.2 at% in CaSrP1.67-BS and CaZnP1.67-BS, respectively, and 10.0 at% of Sr and 10.0 at% of Zn in CaSrZnP1.67-BS (Table 2). Overall, these results indicate that droplet-based microfluidics is a suitable method for producing CaP microparticles with inorganic additives too.

**TABLE 2:** The content of inorganic additives (per total cation content) in CaP microparticles before sintering measured using ICP-MS.

Sample	Sr: (Sr + Zn + Ca) (at%)	Zn: (Sr + Zn + Ca) (at%)
CaSrP1.67-BS	9.292 ± 0.001	-
CaZnP1.67-BS	-	10.207 ± 0.001
CaSrZnP1.67-BS	10.013 ± 0.001	10.017 ± 0.001

### Porosity and surface roughness of CaP microparticles

**FIGURE 7:**
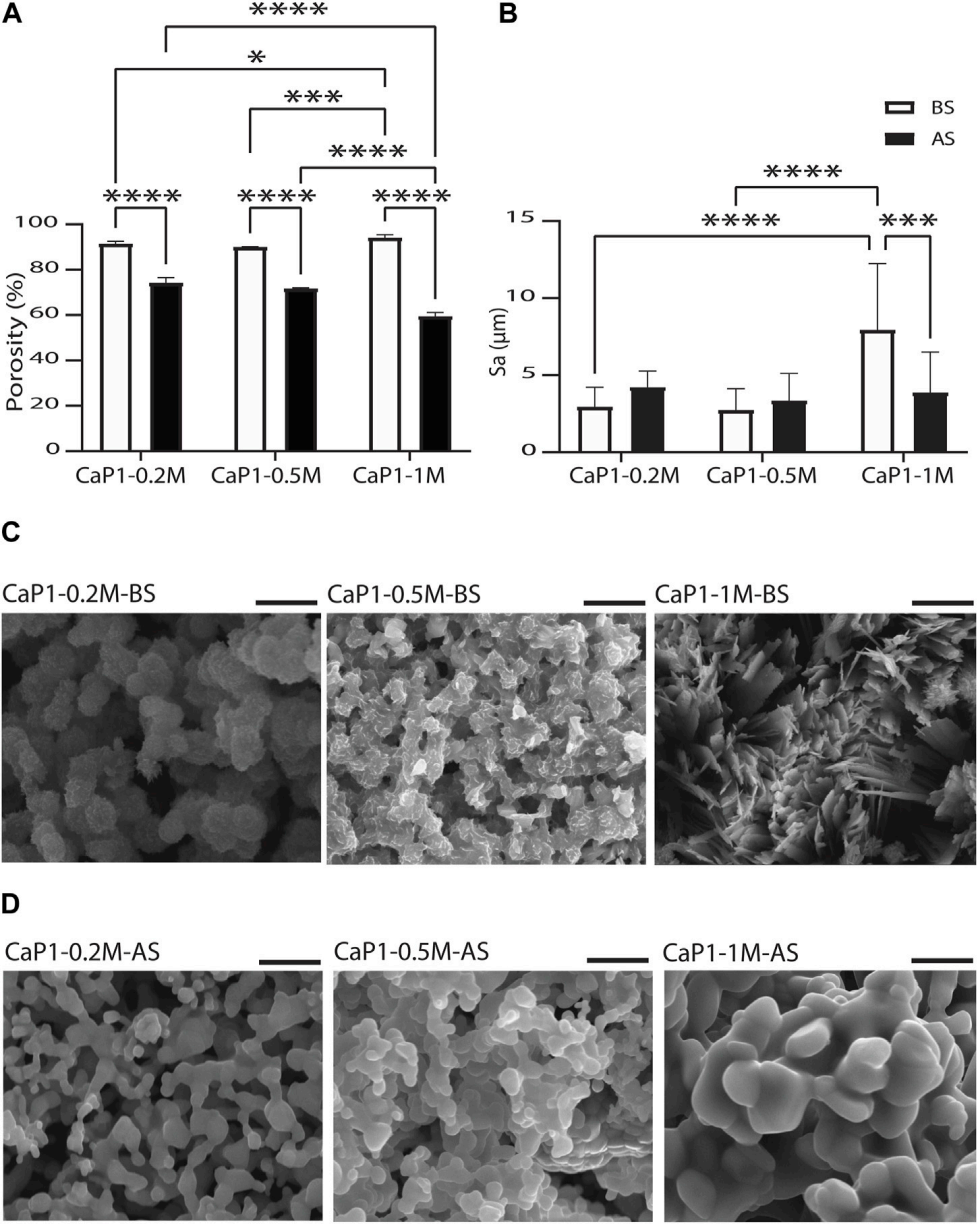
Porosity and surface roughness of CaP microparticles. Quantification of **(A)** microporosity of CaP discs and **(B)** surface roughness levels in CaP microparticles. SEM images of the microstructures of CaP microparticles produced with Ca/P of 1 and different precursor concentrations **(C)** before (BS) and **(D)** after sintering (AS). Scale bars in **(C)** and **(D)**: 5 μm.

The porosity and the surface roughness of CaP microparticles produced using the droplet generator microfluidic chip, and the parameters that may directly affect porosity and roughness were investigated (Figures 7A, B). This may eventually enable the production of CaP microparticles with fine-tuned microstructures. All CaP microparticles showed porous microstructures with pores at micro- and nano-scales (Figures 5B, D). CaP microporosity is closely related to the ceramic grain size and shape. Sintering parameters, including time and temperature, are often used to control the grain size and microporosity of CaPs (AbdulQader et al., 2013; Galván-Chacón and Habibovic, 2017; Ghayor et al., 2020). Other physical and chemical factors during the wet chemical synthesis of CaPs (Lin et al., 2014), including the initial Ca/P ratio and concentration of the precursor solution, synthesis temperature, aging time, and the use of surfactants can also influence their grain morphology and size, and consequently, their microporosity. Therefore, we investigated the effects of a subset of these factors, namely, the precursor concentration and sintering, on the total porosity of discs made out of the microparticles with Ca/P of 1. Prior to sintering, high levels of porosity (≥89.99%) were measured in the discs (Figure 7A). No significant differences were found in the porosity of the discs obtained with microparticle with lower precursor concentration, i.e. 0.2 M (CaP1-0.2M-BS) and 0.5 M (CaP1-0.5M-BS) with respective porosity levels of 91.4% and 90.0%. However, higher precursors concentration, i.e., 1 M (CaP1-1M-BS), led to the significantly higher porosity level of 94.2% in the CaP discs as compared to other concentrations. The CaP (crystal) morphology could plausibly be a prominent cause of these observations, wherein irregularly-packed micron-sized plates in the microstructure of CaP1-1M-BS have led to a higher porosity, as compared to submicron plates packed more densely in the microstructure of CaP1-0.5M-BS and CaP1-0.2M-BS (Figure 7C). The changes in microstructure and crystal morphology of CaPs have been shown to often result in changes in porosity levels or related parameters, such as pore size and (specific) surface area. Such hand-in-hand changes have been in the past observed as a consequence of altering CaP synthesis parameters or sintering conditions, resulting in the formation of CaPs with different phases, and/or microstructure and morphology (Habibovic et al., 2008; Cama et al., 2013; Zhang et al., 2014), as well as due to the addition of cargo, such as drugs, onto the CaPs (Shen et al., 2014).

Sintering of the microparticles resulted in an expected reduction of the total porosity levels of the resulting CaP discs (Figure 7A). Porosity reduction and compactness in the microstructure of solid materials occur during the sintering process due to the fusion of gains or particles, a process that has often been used to increase the mechanical strengths of materials (Fang, 2010). The results also showed that sintered microparticles with higher precursor concentration resulted in CaP discs with lower porosity levels, wherein both CaP1-0.2M-AS and CaP1-0.5M-AS with respective porosities of 74.2% and 71.7%, were significantly more porous than CaP1-1M-AS, with a porosity of 59.4%. Here, all materials presented a rather similar grained morphology with differences in grain size (Figure 7D). These differences were quantifies by manually contouring a total of 80 grains on two SEM images of all CaPs after sintering, and analyzing their area and Feret’s diameter using Fiji software. Both qualitative and quantitative results indicated that CaP1-1M-AS exhibited larger grains than other conditions (Supplementary Figure S3). The larger grain size in this sample may indicate a stronger densification during sintering, in turn leading to lower porosity levels in CaP1-1M-AS as compared to the samples with lower precursor concentration.

It should be noted that the results described above take both the porosity of the microparticles and any space in between the CaP microparticles in the discs into account. To provide further insight on the porosity of microparticles alone, SEM images of sectioned microparticles were assessed. Porosity fraction areas of around 72% and 58% were obtained for CaP1-0.2M-AS and CaP1-1M-AS, respectively, using this method. This was in line with the results measured for CaP discs (Supplementary Figure S4), indicating higher porosity levels for the CaP with smaller grains.

Surface roughness of the CaP microparticles was also evaluated aiming to investigate the influence of parameters such as precursor concentration and sintering on the surface roughness of the CaPs (Figure 7B). To this end, profilometry technique was used to determine the surface arithmetical mean height (Sa), which is an extension of the line arithmetical mean height (Ra), to evaluate the surface roughness of CaP1-0.2M, CaP1-0.5M and CaP1-1M before and after sintering. Here, the morphological aspects seemed to also dominate the surface roughness outcome. Before sintering, no significant difference was observed in the Sa of CaP1-0.2M-BS and CaP1-0.5M-BS. However, in line with its distinct plate-like morphology (Figure 7C), CaP1-1M-BS expectedly presented a significantly higher Sa than its counterparts produced with lower precursor concentrations. After sintering, the Sa values decreased significantly (down to 3.9 μm) in CaP1-1M-AS as expected and due to the substantial morphology changes from a plate-like to a grained one (Figure 7D). In this case, statistically similar values (with slight increases) were observed in the other samples, which experienced changes from globular morphologies to grained ones.

CaP pore size and microporosity, along with the grain size, directly impacts the specific surface area (Habibovic et al., 2006a) and can in turn significantly influence the bioactivity/ion exchange, protein adsorption, cell adhesion and osteogenic-related functions. The significance of total porosity and microporosity for the bone-forming potential of CaPs was investigated previously. For example, Habibovic et al. reported osteoinductivity (i.e., the ability to induce bone formation in an ectopic cite) and improved osteoconductivity (i.e., bone-forming ability in an orthotopic cite) *in vivo*, in BCP particles with higher microporosity level and larger specific surface area (Habibovic et al., 2006b; Habibovic et al., 2008). Yamasaki et al. also showed osteoinductive behavior in porous HA after subcutaneous and intramuscular implantations in dogs, whereas no bone formation was observed in dense HA (Yamasaki and Sakai, 1992). Similarly, (subtle) differences in other physical properties of the surface, such as surface roughness, affect the real area of contact for cells, and can substantially modulate cell-biomaterial interactions, such as cell adhesion and proliferation, and guide cell fate (Ventre and Netti, 2016; Xiao et al., 2020). Deligianni et al. reported that rougher HA discs (Ra = 4.68 μm) enhanced cell adhesion, detachment strength and proliferation of hBMSCs as compared to their smoother counterparts (Ra < 3 μm) (Deligianni et al., 2000). Favorable effects of rougher CaPs on osteogenic differentiation of human and rat osteoblast (-like) cells were also reported (Cairns et al., 2010; Costa et al., 2013). Taken together, the (micro)structural aspects of CaPs, such as porosity, pore size and surface roughness, present strong modulating factors for generating high-performance CaP biomaterials. Here, it is shown that tailored CaPs with different porosity levels can be obtained using the droplet-generating microfluidic device by adjusting the concentration of the precursors and sintering condition. Nonetheless, it is important to note that changes in these parameters can cause changes in the chemical composition of the CaPs too. Further research is required to precisely control the microporosity of microparticles during the droplet microfluidic-based synthesis and to determine the full extent of the roles of these parameters on affecting the interactions of the biological system with the CaPs. Alternatively, controlled microporosity can be introduced into the green body of CaPs by means of the processing method (Galván-Chacón and Habibovic, 2017), for example, by using porogens, or by templating and 3D printing (Wu and Uskoković, 2016; Raja et al., 2022; Mandić et al., 2023). Similarly, using porogens in droplet microfluidic methods can result in microparticles with controlled porosity (Yeh et al., 2023)).

### Degradation of CaP microparticles

It has been shown that different physicochemical properties of CaPs, including chemical composition, crystallinity, microstructure and porosity, influence their degradation and ion exchange behavior (Pan et al., 2009; Schaefer et al., 2011; Wu and Uskoković, 2016). The bioactivity of CaPs is often measured based on the amount of new CaP deposited/precipitated onto their surface in Ca^2+^- and Pi-rich medium. Here, the degradation of CaP microparticles was investigated in cell culture medium, and the Ca and P levels were measured for up to 15 days using ICP-MS.

**FIGURE 8:**
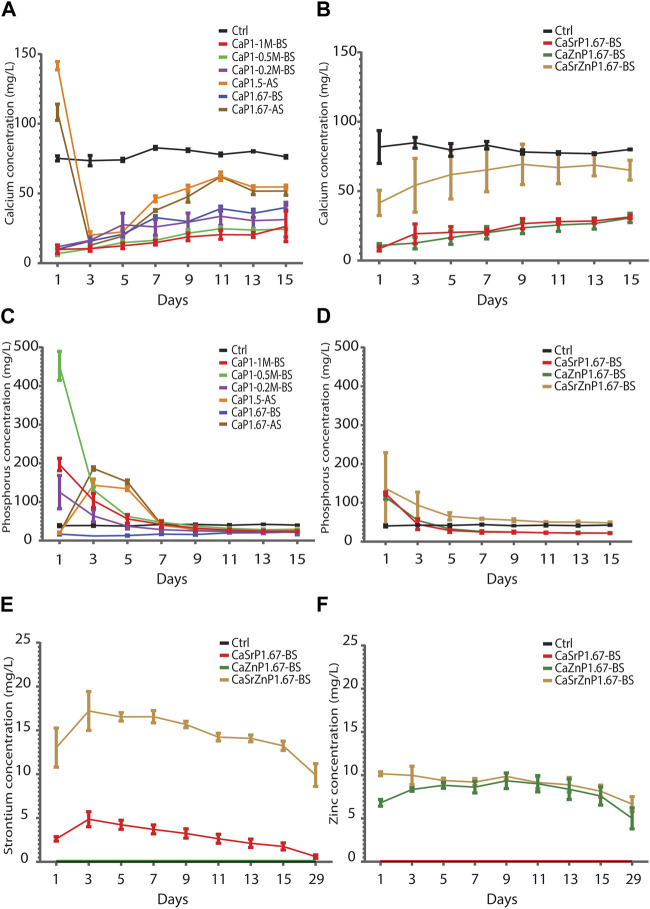
Degradation of CaP microparticles over time in cell culture medium. **(A,B)** Ca and **(C,D)** P levels in cell culture medium incubated in physiological conditions with CaP microparticles **(A,C)** without and **(B,D)** with inorganic additives. **(E)** Sr and **(F)** Zn levels in cell culture media incubated with CaP microparticle containing inorganic ions.

The Ca levels in the cell culture medium control without CaP microparticles remained rather steady over time at 78-84 mg/L. The results showed that sintered CaP microparticles (CaP1.5-AS and CaP1.67-AS) had higher Ca levels at day 1 compared to non-sintered CaPs and cell medium control. This initial surge in the Ca levels may plausibly be due to the presence of trace amounts of Ca salts more soluble than HA and β-TCP, which may have formed in the samples during sintering (Döbelin et al., 2020). At day 3, the Ca levels decreased below that of the control, indicating the uptake of Ca^2+^ and marking the onset of the CaP precipitation (Reynaud et al., 2021). The non-sintered CaPs showed a substantially lower Ca levels in cell medium compared to the control at all time points, in a generally ascending fashion (Figure 8A). This indicates the Ca^2+^ uptake and the CaP precipitation starting from the first time point. The P level in the sintered samples showed an abrupt increase at day 3, followed by a gradual decrease to lower levels than those in the control samples at the later time points. The P amounts in the samples CaP1-0.2M-BS, CaP1-0.5M-BS and CaP1-1M-BS were higher than the control at early time points (day 1 to day 3), with CaP1-0.5M-BS having the highest levels. This again may be due to the presence of small amount of highly soluble phosphate phases. P levels in these samples decreased over time to reach similar amounts as the control at day 7, after which the P levels stabilized at lower levels than the control, again indicating CaP precipitation in these samples (Figure 8C). Overall, these results suggest the precipitation of CaP in all the samples and over time, particularly from day 7 on.

Similarly, at all time points, lower Ca levels in cell medium were observed for CaSrP1.67-BS, CaZnP1.67-BS and CaSrZnP1.67-BS as compared to the control, with the highest Ca levels detected for CaSrZnP1.67-BS (Figure 8B). An increase of P level at day 1 as compared to the control was observed and the P level for the sample with Sr and Zn co-addition was higher than that in the samples with individual additions of Sr or Zn (Figure 8D), following the same trend as their Ca levels. The sustained release of Sr and/or Zn from CaPs with inorganic additive(s) was observed up to 29 days of incubation in cell culture medium. A higher level of released Sr was seen in CaSrZnP1.67-BS as compared to CaSrP1.67-BS. The Zn release in the samples with individual and dual addition of ions appeared similar though (Figures 8E, F). The higher Sr release observed in CaSrZnP1.67-BS can be explained by its lower crystallinity compared to CaSrP1.67-BS, increasing its dissolution in aqueous environment and leading to a higher dissolution rate in this sample. The presence of inorganic additives has been previously shown to alter the solubility behavior of CaPs (Ullah et al., 2020). For example, addition of ions to CaPs was shown to change their crystallinity, and consequently led to faster dissolution of the CaPs (Dasgupta et al., 2010).

**FIGURE 9:**
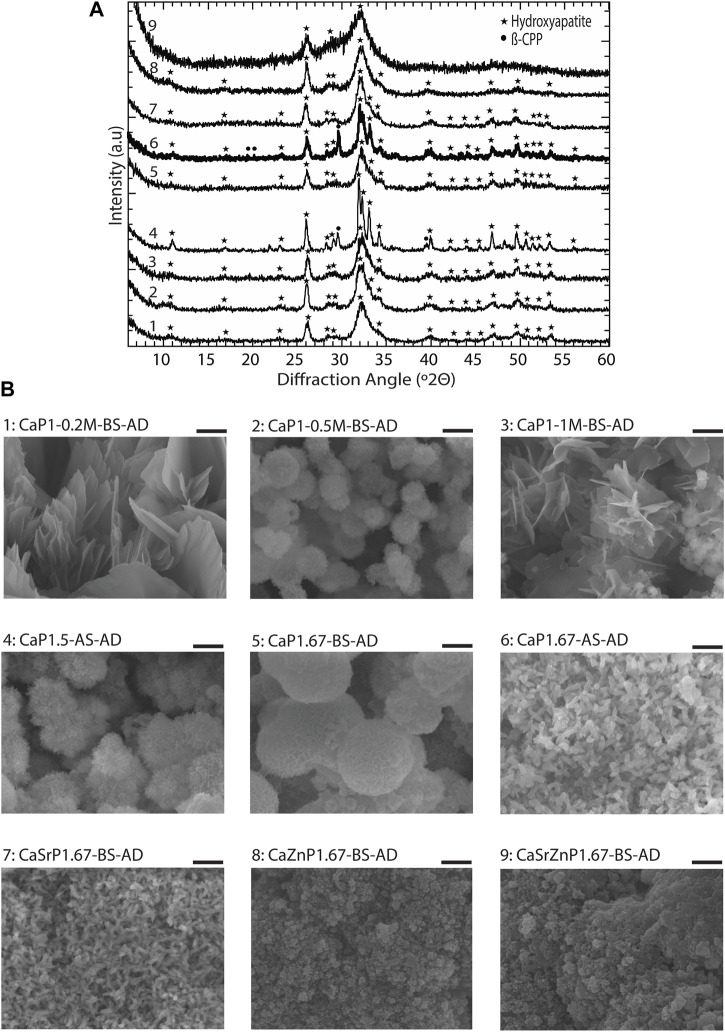
Biomimetic HA formation on CaP microparticles in cell culture medium. **(A)** XRD patterns and **(B)** SEM images of CaP microparticles after 29 days of incubation in cell culture medium under physiological conditions. Scale bars: 2 μm. AD indicates after degradation.

The chemical composition and morphology of CaP microparticles after incubation in cell culture medium was also investigated. The XRD patterns of all CaP microparticles after 29 days of incubation in cell media showed the presence of CaP phases predominantly composed of HA, indicating the HA precipitation in the microparticles (Figure 9A). This suggest the high bioactivity levels of CaP microparticles (Carino et al., 2018). Sharper HA peaks, indicating higher crystallinity, were observed in the sintered CaPs (i.e., CaP1.5-AS and CaP1.67-AS). On the other hand, a closer-to-amorphous apatitic phase in the case of CaSrZnP1.67-BS was detected. The SEM characterization (Figure 9B) showed that the new CaPs formed on the surface of microparticles has morphologies previously observed in (biomimetic) CaPs formed in simulated body fluids (Barradas et al., 2013; Guttenplan et al., 2021b). No specific trends in the surface roughness levels could be observed (Supplementary Figure S5). However, overall, the different behaviors of the CaPs in cell medium suggests that their interactions with cells may consequently differ.

## Conclusions and perspectives

This study aimed to optimize the different steps of a droplet microfluidic-based process, including droplet generation, in-droplet CaP synthesis, purification and sintering, for obtaining a library of CaP microparticles with fine-tuned properties. A flow-focusing microfluidic droplet generator was used for the rapid production of large quantities of monodisperse water-in-oil droplets, in which the water phase consisted of Ca and P precursors. CaP microparticles were mineralized inside the droplets by increasing the in-droplet pH with a basic solution diffused through the oil shell. The optimized purification consisted of several washing steps with diethyl ether aiming to render the microparticles free of oil contamination. By introducing small adjustments in the process, including the Ca and Pi precursors concentrations and molar ratios, aging time and post-synthesis sintering, in-droplet synthesis of several members of the CaP family, including monetite, brushite, low- and high-crystalline HA, β-TCP and β-CPP, was achieved. By introducing third and fourth ion precursor (i.e., Sr and/or Zn) into the water phase, CaP microparticles with the addition of the respective inorganic additives were produced. In addition, the microparticles presented different chemical and microstructural properties, such as degradation behavior, surface morphology, porosity and roughness, all of which can be employed to orchestrate cell-biomaterial interactions.

The droplet microfluidic system led to the development of an easily-adjustable and controllable chemical process with minimal waste of resources. This process can be in the future used to attain designer (inorganic) microparticles using upgraded microfluidic droplet generators for example, double emulsion droplet generators. In addition, the presented microfluidic system allows for easy parallelization of the process, which in turn can facilitate the high-throughput synthesis and pre-clinical screening of various CaP biomaterials while consuming fewer resources as compared to the conventional one-experiment-for-one-material methods, and the scaled-up production of the optimized CaP formulations for bone regeneration applications. Prospectively, automation of the process, including droplet-generation, in-droplet mineralization, purification, characterization and quality control steps, is another feature that can be integrated into the microfluidic-based method described here, which can also enable both high-throughput and scaled-up production of CaP-based biomaterials.

## Data Availability

The raw data supporting the conclusions of this article will be made available by the authors, without undue reservation.
