# Supplementary material for: Optimization of a tunable process for rapid production of calcium phosphate microparticles using a droplet-based microfluidic platform
Source: Front Bioeng Biotechnol. 2024 Mar 27;12:1352184. doi: 10.3389/fbioe.2024.1352184 (PMC11004461; doi:10.3389/fbioe.2024.1352184)
Supplement: Supplementary file 1 [file DataSheet1.docx]

**Optimization of a tunable process for rapid production of calcium phosphate microparticles using a droplet-based microfluidic platform**

Y. Alaoui Selsouli^1^, H.S. Rho^1^, M. Eischen-loges^1^, V.P. Galván-Chacón^1^, C. Stähli^2^, Y. Viecelli^2^, N. Döbelin^2^, M. Bohner^2^, Z. Tahmasebi Birgani^1^, P. Habibović^1*^

^1^Department of Instructive Biomaterials Engineering, MERLN Institute for Technology-Inspired Regenerative Medicine, Maastricht University, P.O. Box 616, 6200 MD Maastricht, The Netherlands.

^2^RMS Foundation, Bischmattstrasse 12, 2544 Bettlach, Switzerland.

* Corresponding authors; email: [p.habibovic@maastrichtuniversity.nl](mailto:p.habibovic@maastrichtuniversity.nl)

**Supplementary material**

**
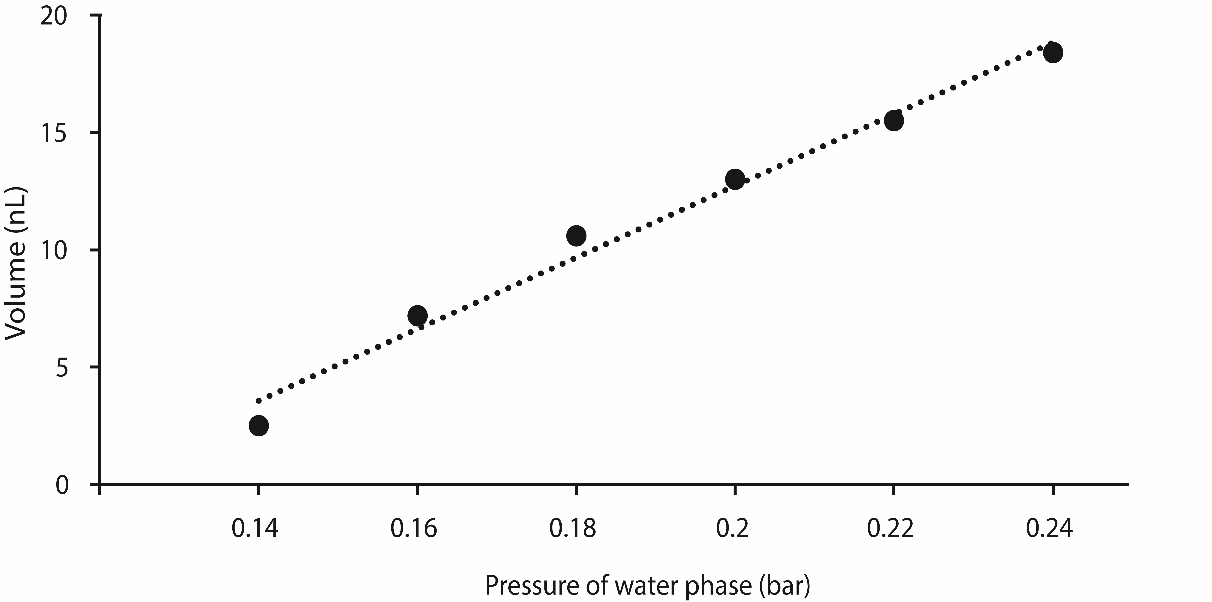
**

Supplementary Figure S1. Standard curve of the microdroplet volume based on the pressure of the water phase.


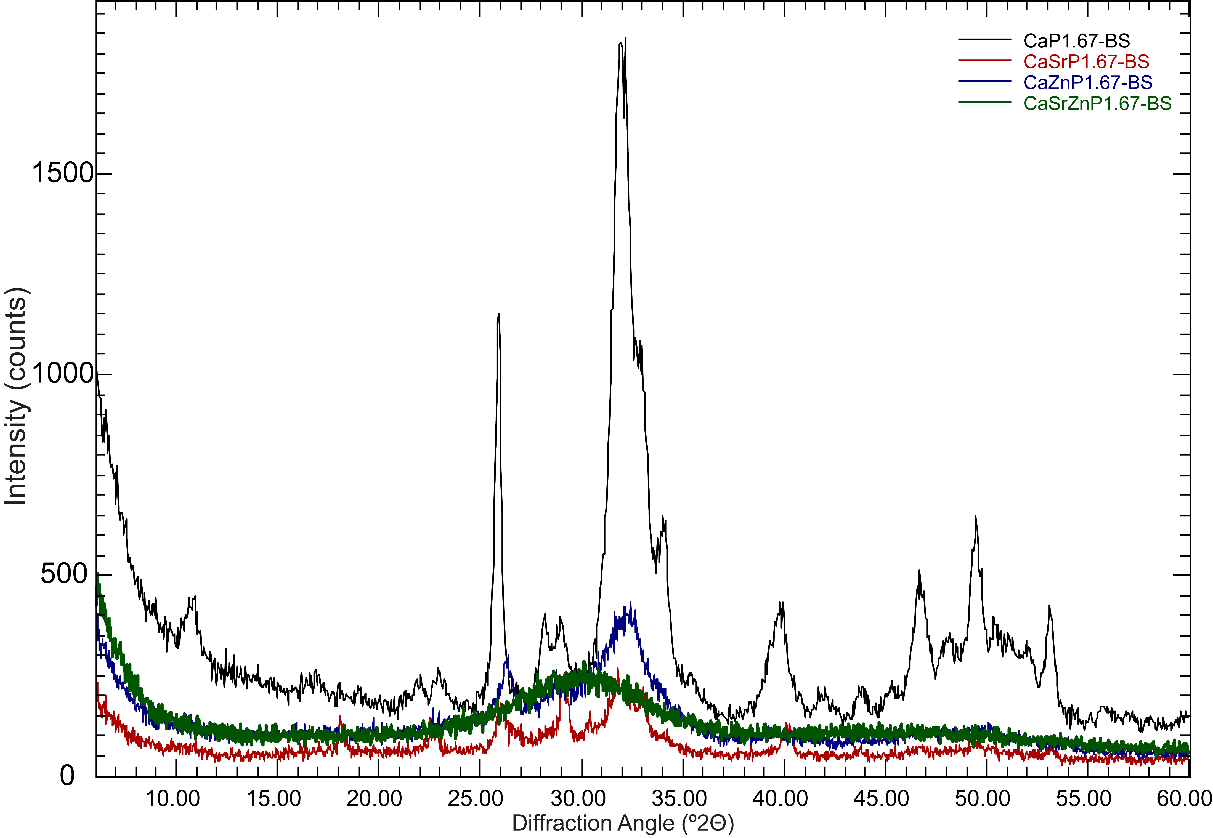


Supplementary Figure S2. Comparison of (the intensity and width of the peaks) in the XRD patterns of CaPs with Ca/P of 1.67, with and without inorganic additives, indicating lower crystallinity of CaP phases with inorganic additives.


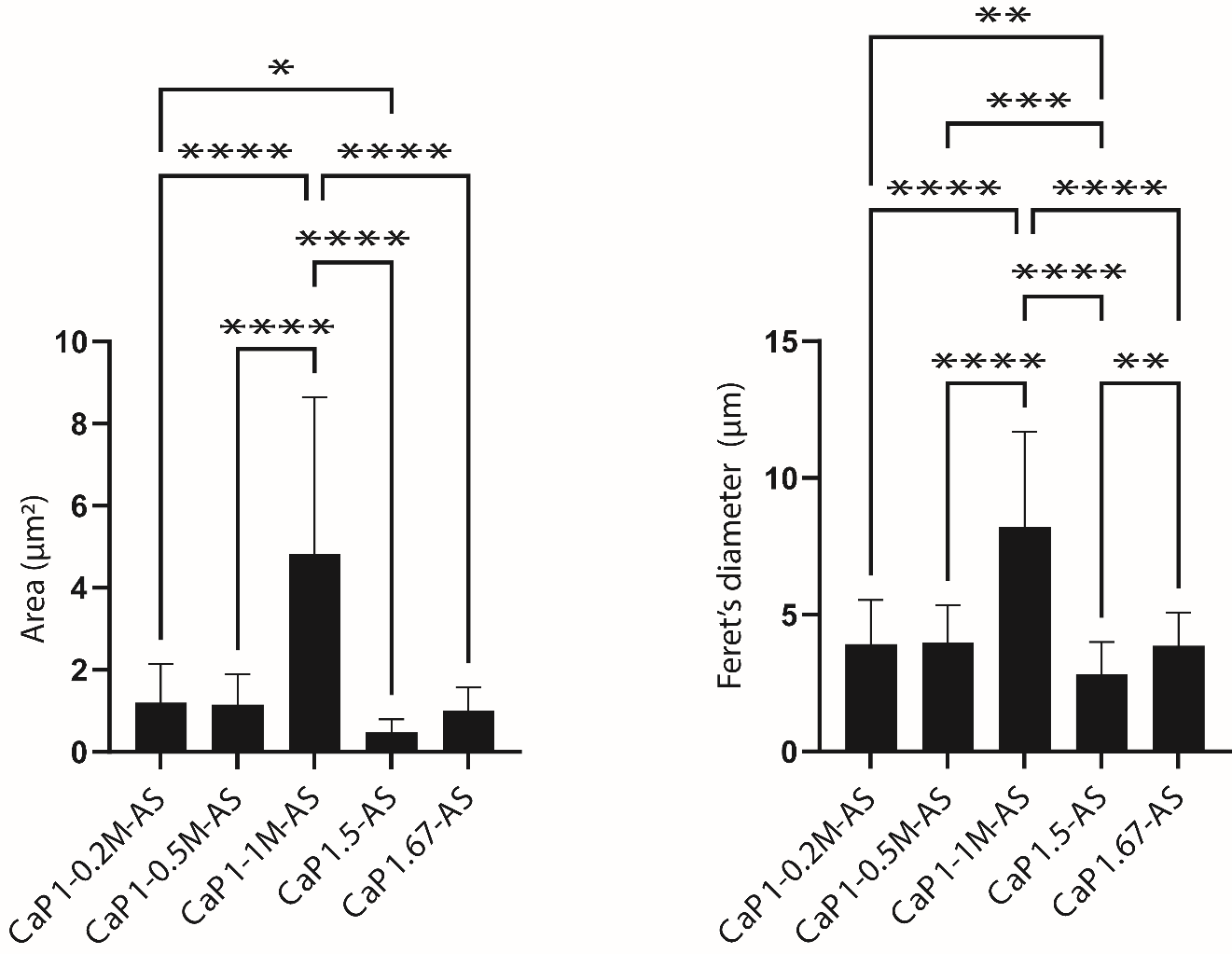


Supplementary Figure S3. Average area and average Feret’s diameter of selected grains of sintered CaPs, indicating the larger grain size in CaP1-1M-AS compared to other CaPs with different Ca/P ratios, as well as compared to those with the same Ca/P ratio and different precursor concentrations.


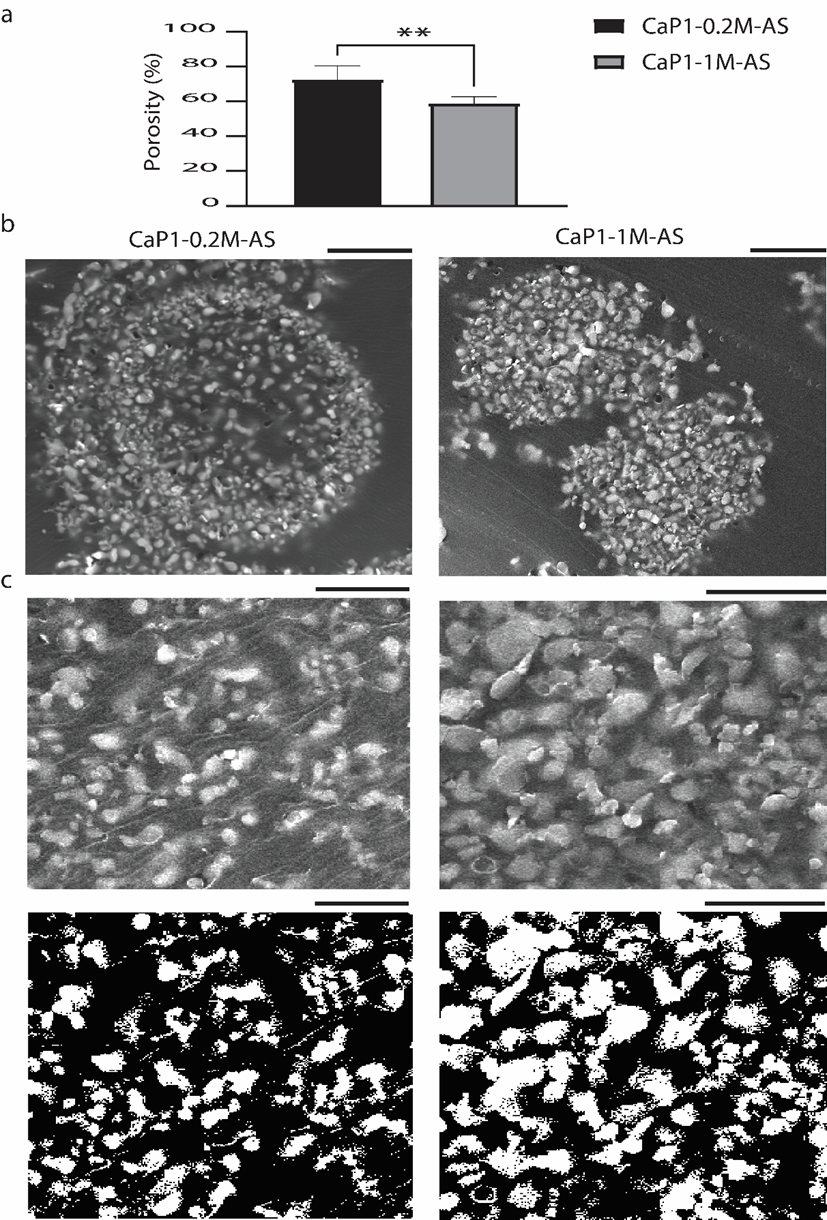


Supplementary Figure S4. Porosity of CaP microparticles. (a) Quantification of the microporosity of CaP1-0.2M-AS and CaP1-1M-AS microparticles by analyzing the SEM images of the sectioned microparticles (n=5). SEM images of (b) sectioned microparticles (scale bars: 10 μm), and (c) examples of regions of interest used for the microporosity quantification in normal (top) and binary (bottom) modes (scale bars: 5 μm).


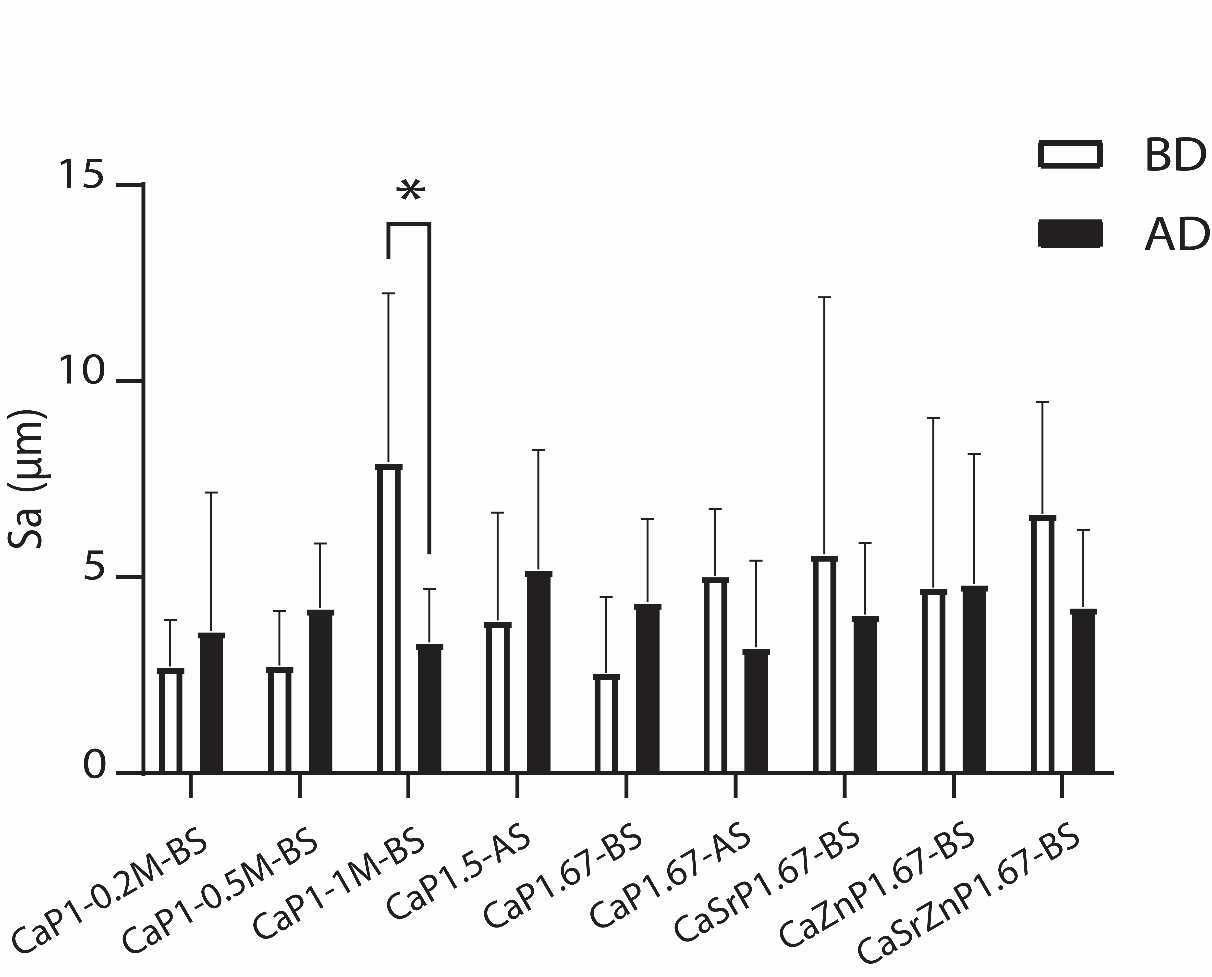


Supplementary Figure S5. Changes in the surface roughness of CaP microparticles after 29 days of incubation in cell culture media. BD and AD respectively indicate before and after degradation.

Supplementary Table S1. The content of inorganic additives (per total cation content) in CaP microparticles before sintering measured using EDS.

| **Sample** | **Sr:(Sr+Ca+Zn)**  **(at%)** | **Zn:(Sr+Ca+Zn)**  **(at%)** |
| --- | --- | --- |
| CaSrP1.67-BS | 11.46 ± 0.03 | - |
| CaZnP1.67-BS | - | 10.82 ± 0.04 |
| CaSrZnP1.67-BS | 9.82 ± 0.02 | 9.14 ± 0.04 |
